# Cardiovascular disease in COVID-19: a systematic review and meta-analysis of 10,898 patients and proposal of a triage risk stratification tool

**DOI:** 10.1186/s43044-020-00075-z

**Published:** 2020-07-13

**Authors:** Sara Momtazmanesh, Parnian Shobeiri, Sara Hanaei, Hani Mahmoud-Elsayed, Bharat Dalvi, Elaheh Malakan Rad

**Affiliations:** 1 Tehran University of Medical Sciences, Tehran, Iran; 2 Scientific Education and Research Network (USERN), Tehran, Iran; 3 Research Center for Immunodeficiencies, Tehran University of Medical Sciences, Tehran, Iran; 4 Cardiology Department, Queen Elizabeth Hospital, University Hospitals Birmingham NHS Foundation Trust, Birmingham, UK; 5 Glenmark Cardiac Centre, Mumbai, India; 6 Department of Pediatric Cardiology, Children’s Medical Center (Pediatric Center of Excellence), Tehran University of Medical Sciences, Tehran, Iran

**Keywords:** COVID-19, Cardiovascular disease, Troponin, Interleukin-6, Hypertension

## Abstract

**Background:**

Coronavirus disease 2019 (COVID-19) pandemic has drastically affected global health. Despite several studies, there is yet a dearth of data regarding the mechanisms of cardiac injury, clinical presentation, risk factors, and treatment of COVID-19-associated cardiovascular disease. This systematic review and meta-analysis is aimed at defining the clinical, electrocardiographic, and pathologic spectrum of cardiovascular disease (CVD), frequency of elevated cardiac and inflammatory biomarkers, and their frequency and relationship with severity of the disease and mortality in COVID-19 patients and to develop a triage risk stratification tool (TRST) that can serve as a guide for the timely recognition of the high-risk patients and mechanism-targeted therapy. We conducted an online search in databases of PubMed and Embase to identify relevant studies. Data selection was in concordance with PRISMA guidelines. Results were presented as pooled frequencies, odds ratio, standardized mean difference (SMD), and forest and funnel plots.

**Results:**

We gathered a total of 54 studies and included 35 of them in our meta-analysis. Acute cardiac injury occurred in more than 25% of cases, mortality was 20 times higher, and admission to intensive care unit increased by 13.5 times. Hypertension was the most common pre-existing comorbidity with a frequency of 29.2%, followed by diabetes mellitus (13.5%). The deceased group of patients had higher cardiac and inflammatory biomarkers, with statistically significant SMD, compared with survivors. Pediatric patients were predominantly mildly affected. However, less frequently, the presentation was very similar to Kawasaki disease or Kawasaki shock syndrome. This latter presentation hass been called as multisystem inflammatory syndrome in children (MIS-C).

**Conclusions:**

There is a wide spectrum of cardiac involvement in COVID-19 patients, and hence a Triage Risk Stratification Tool can serve as a guide for the timely recognition of the high-risk patients and mechanism-targeted therapy.

## Background

Coronavirus disease 2019 (COVID-19) pandemic has drastically affected the global health and as of 15th May 2020, resulted in 4,248,389 million confirmed cases and a death toll of 294,046 worldwide [1].

This disease has presented with a heterogeneous clinical course, ranging from asymptomatic carrier state to a lethal outcome with multi-organ failure and with a wide variety of case fatality rates ranging from 0.7 to 67% [2–4]. Although the respiratory tract is the most commonly involved organ system in this disease, other organs and particularly the heart are also affected with a negative impact on outcome [5]. Furthermore, pre-existing cardiovascular disease (CVD) can affect severity and mortality of these patients. Despite myriads of studies investigating cardiovascular diseases in patients with COVID-19, there are still numerous unanswered questions, most importantly a triage risk stratification tool (TRST) that allows timely recognition of high-risk patients and well-timed delivery of risk-level-appropriate, patient-tailored, and pathophysiological-targeted treatment [6].

The aims of this systematic review and meta-analyses were (1) to calculate pooled frequency of newly developed and pre-existing CVD, hypertension, diabetes mellitus, cardiac symptoms as the initial presentations of COVID-19, elevation of cardiac and inflammatory biomarkers, acute hepatic, and renal injury; (2) to investigate association of newly developed and pre-existing CVD (including any acquired cardiac disease, encompassing ischemic and non-ischemic cardiomyopathies, or congenital heart disease) hypertension, and elevated cardiac and inflammatory biomarkers with severity of the disease and mortality; (3) to define the clinical spectrum and mechanisms of the newly developed cardiovascular diseases in the pediatric and adult population, the spectrum of newly developed arrhythmias and electrocardiographic changes and the pathologic findings of cardiac autopsies; and (4) to propose a TRST for timely detection and appropriate pathophysiologically targeted treatment of high-risk COVID-19 patients with associated CVD.

## Methods

### Literature search and selection criteria

We conducted an online search in databases of PubMed and Embase on 21st April 2020 to identify relevant studies. The search terms included “COVID-19,” and “cardiovascular diseases,” and other relevant or equivalent terms. We provided our search strategy in Supplementary Material 1 (S1). To retrieve additional eligible studies, we also traced the reference list of the retrieved papers and relevant reviews.

Studies were included if (1) they had reported associated cardiovascular diseases in COVID-19 patients, (2) assessed levels of cardiac biomarkers in COVID-19 patients, and (3) were original peer-reviewed studies (except for one study that we used in our qualitative analysis and was retrieved from MedRxiv) [7]. We did not apply any limitation on language or publication date. Studies were included in our quantitative analysis if they had a sample size of equal to or larger than ten. The rest of the articles, including case reports, case series, and studies investigating pathological features of the heart tissue, were assessed in the qualitative analysis. We excluded review articles.

Data selection was in concordance with the Preferred Reporting Items for Systematic Reviews and Meta-Analyses (PRISMA) guidelines [1]. Two authors (SM and PS) independently assessed the eligibility of the retrieved references. In case of disagreement, EMR made the final decision.

### Data extraction

Three reviewers (SM, PS, and EMR) extracted (1) characteristics of the sample (age, gender, previous cardiovascular comorbidities, diabetes, and hypertension); (2) incidence of cardiovascular diseases; (3) levels of cardiac biomarkers, including troponins, N-terminal-pro B-type natriuretic peptide (NT-pro BNP), myoglobin, creatine kinase (CK), creatine kinase-MB (CK-MB), and lactate dehydrogenase (LDH) and inflammatory biomarkers including d-dimer, C-reactive protein (CRP), erythrocyte sedimentation rate (ESR), ferritin, interleukin-6 (IL-6), and tumor necrosis factor-alpha (TNF-α); and (4) frequency of acute hepatic injury and acute kidney damage.

### Risk of bias assessment

To critically appraise the included studies, we implemented the Newcastle–Ottawa scale (NOS) [8]. The possible scores of this scale range from 0 to 9. Studies with a score of seven to nine, four to six, and zero to three were classified as studies with low, moderate, and high risk of bias, respectively.

### Statistical analysis

We used OpenMeta Analyst [9] version 1.15.14 and RevMan version 5.3 [10]. Using forest plots, we illustrated the results of the analyses. We used funnel plots to illustrate the publication bias. The odds ratio (OR) were calculated to compare frequencies of acute cardiac injury, hypertension, and pre-existing cardiovascular diseases, between the “deceased” and the “recovered” patients and also between patients with “severe” and “non-severe” manifestations. We used standardized mean difference (SMD) to compare levels of cardiac troponin, CK, NT-pro BNP, myoglobin, LDH, CRP, ESR, ferritin, IL-6, and LDH between the “deceased” and “recovered” patients and levels of CRP, LDH, CK, and CK-MB between the “severe” and “non-severe” cases.

We used the *I*^2^ index to assess heterogeneity between studies. The *I*^*2*^-indices of 0–25%, 26–75%, and 75–100% represented low, moderate, and high degrees of heterogeneity, respectively [11]. We utilized fixed effects models if the results were homogeneous (*I* < 50% and *P* > 0.05) and random effect models if these results were heterogeneous (*I* ≥ 50% or *P* ≤ 0.05) [12].

To convert median and inter-quartile range (IQR) to mean and standard deviation (SD), we used statistical methods suggested by Luo et al. [13] and Wan et al. [14]. For one study which did not report the first and third quartiles of its data, we assumed the mean equal to the median and the standard deviation (SD) equal to IQR divided by 2 [6].

Our analysis of the comparison of patients with severe to those with non-severe COVID-19 included two types of categorization, namely “ICU” versus “non-ICU” groups and “severe” (defined as respiratory rate ≥ 30 times/min, oxygen saturation at resting state ≤ 93%, partial pressure of arterial oxygen to fraction of inspired oxygen ratio < 300) versus “non-severe” (presented as without pneumonia or with mild pneumonia) categories. We also calculated the overall effect of each parameter on both groups (severe or ICU group and non-severe or non-ICU group) as a whole. In the series of studies with “ICU” versus “non-ICU” groups, we did not include the study performed by Du et al. [15], since they had clearly stated that all of their patients had an indication for ICU admission, but they could not admit them into ICU just because of a shortage of resources. Regarding study of Han et al. in which they had classified their patients into three subgroups as mild, severe, and critical, we merged the severe and critical groups. Additionally, we did not include Wan et al.’s [16] study in our subgroup analysis comparing acute cardiac injury in patients with severe and non-severe disease because their definition of acute cardiac injury was not precisely defined.

In the pooled frequency analysis of patients with higher levels of CRP, ESR, ferritin, IL-6, NT-pro BNP, d-dimer, LDH, cardiac troponins, myoglobin, CK, and CK-MB, we only included the studies which had indicated the number of patients with elevated levels of these biomarkers based on their laboratory cut-off values.

## Results

We present our findings in two sections: “Meta-analyses” and “Systematic review” sections

### Meta-analyses

#### Study selection

**Fig. 1:**
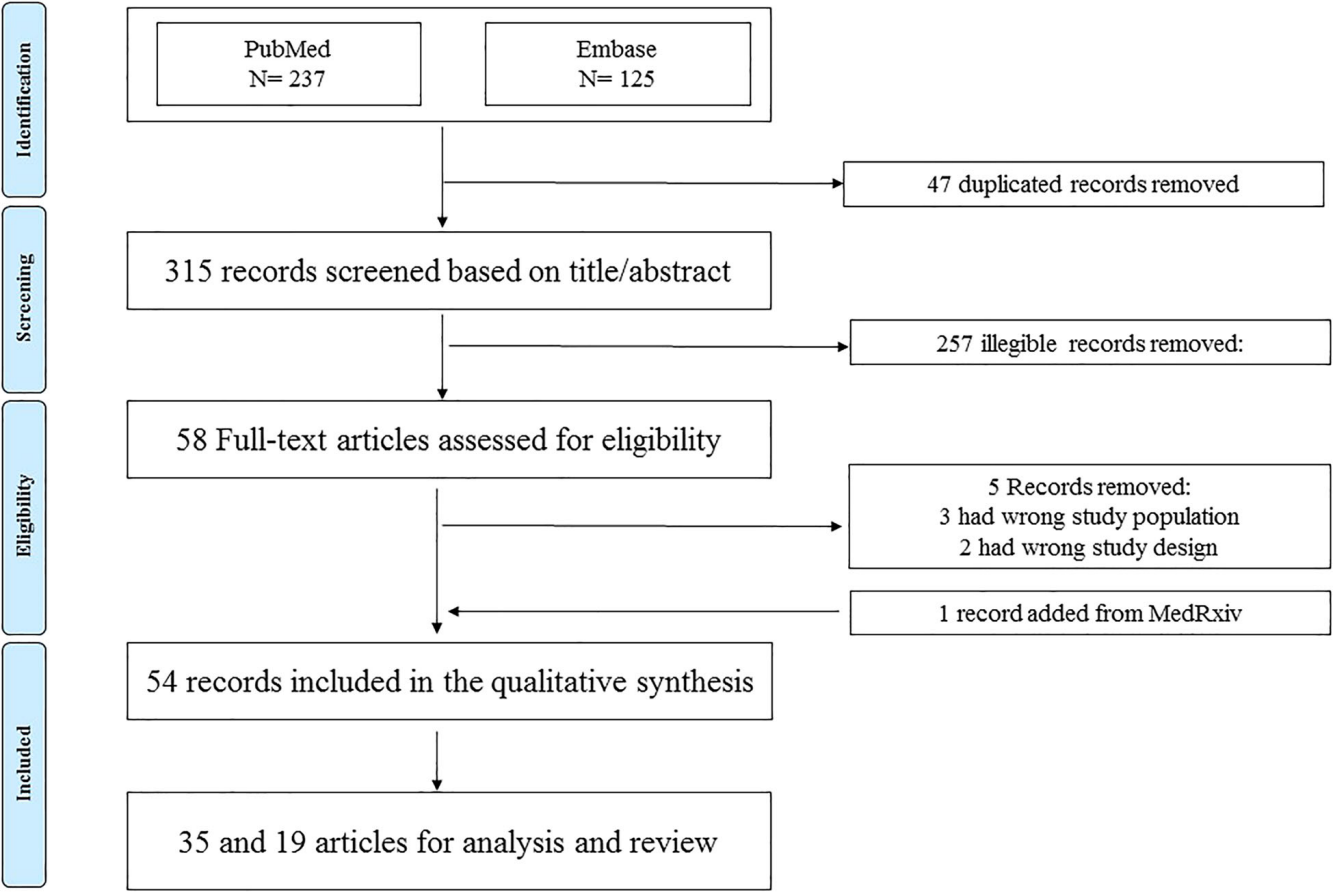
PRISMA Flowchart of data selection

Figure 1 depicts the detailed process of data selection. Three-hundred and sixty-two (362) studies were retrieved in our initial search of PubMed and Embase, 315 of which remained after removing duplicates. In title/abstract screening, 257 papers did not meet our inclusion criteria and were excluded. Five of the studies entering full-text screening had either wrong study design or study population and were excluded. We also added one pre-print study regarding cardiac pathological findings from MedRvix [7]. We included a total of 54 studies in our qualitative synthesis, 35 of which were contained in our meta-analysis [3, 15–47].

#### Features of the included studies

Of the 54 selected studies for the review, 19 records were case reports, case series, or pathological reports. Among studies included in our meta-analysis, nine did not categorize their samples into subgroups, and the rest had a subgroup classification (Tables 1 and 2 in the Supplementary Materials 2 and 3 (S2 and S3). Five studies grouped their patients based on whether they survived or not, three depending on whether they had myocardial injury or high levels of cardiac biomarkers (troponin T or NT-pro BNP), four according to whether patients needed ICU admission or not, and eight had divided their patients based on the severity of manifestations. Three studies used a combination of these classifications and divided their patients into four or more subgroups. Moreover, in three remaining studies, age, the time elapsed from the onset of symptom, or requirement of supplemental oxygen were used to stratify patients.

**Table 1:** The summary of the pooled analysis of frequency of chest pain/chest tightness and palpitation as one of the initial manifestations, newly developed and pre-existing cardiovascular disease, and elevation of cardiac and inflammatory biomarkers in patients with COVID-19

Condition	Number of included studies	Total study population (event) number	Pooled estimated prevalence (95% CI)	Test of heterogeneity	
				***I***^**2**^	***p***
Chest pain/chest tightness and palpitation as one of the initial manifestations					
Chest pain/chest tightness	6	1599 (387)	21.8% (8.5%, 35.0%)	99%	<0.001
Palpitation	2	362 (34)	9.1% (6.2%, 12.1%)	19%	<0.001
Newly developed					
Arrhythmia	4	444 (93)	26.1% (5.9%, 46.4%)	97%	<0.001
Acute cardiac injury	16	2647 (671)	25.3% (19.5%, 31.1%)	93%	<0.001
Heart failure	2	367 (87)	23.7% (19.3%, 28.0%)	0%	<0.001
Pre-existing conditions					
Hypertension	30	10898 (3165)	29.2% (24.7%, 33.6%)	96%	<0.001
Cardiovascular diseases	27	7108 (699)	12.6% (10.0%, 15.2%)	95%	<0.001
Diabetes	30	10806 (1158)	13.5% (11.5%, 15.4%)	89%	<0.001
Heat failure	6	1774 (89)	6.5% (3.3%, 9.7)	95%	<0.001
Elevation of cardiac and inflammatory biomarkers					
Interleukin- 6	3	574 (391)	65.9% (55.2%, 76.5%)	86%	<0.001
CRP	12	3227 (2008)	75.4 % (66%, 84%)	99%	<0.001
ESR	3	767 (547)	71.8% (59.0%, 84.6%)	92%	<0.001
serum ferritin	3	538 (375)	70.3% (61.1%, 79.6%)	81%	0.006
NT-pro BNP	7	1047 (311)	46.5% (28.9%, 64.2%)	98%	<0.001
D-Dimer	8	4789 (2050)	41.5% (31.0%, 52.1%)	97%	<0.001
Lactate dehydrogenase	9	2026 (774)	41.0% (28.8%, 53.2%)	97%	<0.001
Cardiac Tn (I or T)	10	1718 (366)	25.3% (17.6%, 33.1%)	94%	<0.001
myoglobin	5	1076 (176)	19.1% (11.6%, 26.6%)	91%	<0.001
Creatine kinase	10	1617 (230)	15.9% (10.5%, 21.3%)	90%	<0.001
Creatine kinase-MB	2	382 (302)	66.2% (6.9%-100.0%^a^)	99%	<0.001
TNF-α	2	275(472)	58.3% (53.8%-62.7%)	0%	0.7

*Abbreviations*: *NT-pro BNP* N-terminal pro B-type natriuretic peptide, *CRP* C-Reactive Protein, *Tn* troponin, *TNF-α* Tumor Necrosis Factor-α

^a^Due to the high heterogeneity the upper limit of 95% CI was higher than 100%

#### Risk of bias assessment

Of the 35 studies we evaluated in the risk of bias assessment, 30 studies had a low risk of bias, while five records had a moderate risk of bias. None of the included studies were assessed as a study with a high risk of bias. The table of risk of bias assessment is available in Supplementary Material 4 (S4).

#### Results of the meta-analyses

Table 1 illustrates the summary of the results of the pooled analysis of the frequency of chest pain/chest tightness and palpitation as one of the initial manifestations, newly developed and pre-existing cardiovascular disease, and elevation of cardiac and inflammatory biomarkers. The forest plots are available in Supplementary Material 5 (S5).

##### Newly developed cardiovascular diseases or related symptoms

Acute cardiac injury, with an estimated pooled frequency of 25.3% (95% CI 19.5–31.1%), was the most commonly reported cardiac complication of COVID-19. Although the pooled estimated frequency of arrhythmia was slightly higher than acute cardiac injury (26.1%), it was only reported in four studies, and the 95% confidence interval (CI) was equal to 2.5–51.5%. Notably, there was significant heterogeneity in the estimates of newly developed acute cardiac injury and arrhythmia (*I*^2^ = 93%). Moreover, two studies with a total number of 367 patients had assessed newly developed heart failure due to COVID-19 infection, and the pooled frequency was calculated at 23.7% (CI 19.3–28.0%).

Among the cardiac manifestations as the initial presentation of COVID-19, frequency of chest pain or chest tightness and palpitation were investigated. A total of six studies had reported chest pain or chest tightness in COVID-19 patients. Our pooled frequency analysis showed that this presentation was observed as an initial manifestation in approximately one-fifth (21.8%, 95% CI 19.3–28%) of patients. Additionally, two other studies had reported palpitation as the initial presentation, the estimated pooled frequency of which equaled to 9.1% (95% CI 6.2–12.1%).

##### Pre-existing cardiovascular diseases, diabetes, and hypertension

Hypertension was the most common pre-existing comorbidity among COVID-19 patients with a pooled frequency of 29.2% (95% CI 24.7–33.6%), followed by diabetes with a pooled frequency of 13.5% (95% CI 11.5–15.4%). Overall, fewer than one-fifth of patients had pre-existing cardiovascular diseases. The pooled frequency of cardiovascular diseases was estimated at 12.6% (95% CI 10.0–15.2%). Additionally, our analysis on the pooled frequency of heart failure using data of five studies, which had reported pre-existing heart failure, showed a pooled frequency of 6.3% (95% CI 2.9–9.8%). There was significant heterogeneity in the estimates of pre-existing cardiovascular diseases and hypertension (*I*^2^ ≥ 95%).

##### Elevated levels of cardiac and inflammatory biomarkers

**Fig. 2:**
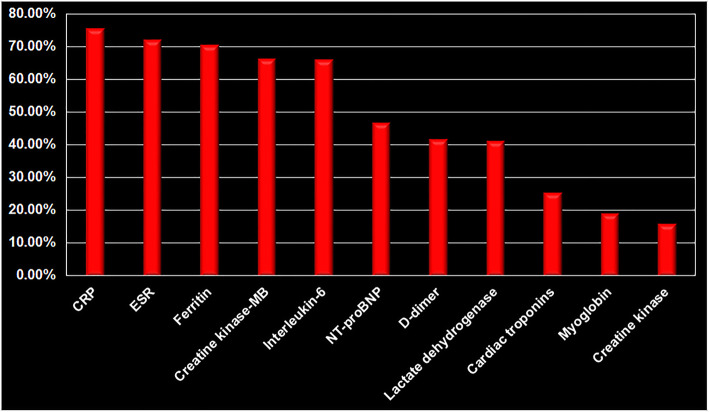
Pooled frequency of elevated cardiac and inflammatory biomarkers

Figure 2 illustrates the pooled frequency of elevation of cardiac and inflammatory biomarkers. Among the biomarkers we investigated, ESR and CRP were the most commonly elevated biomarkers. The estimated pooled frequencies of patients with elevated ESR and CRP were 71.8% (95% CI 59.0–84.6%) and 75.4% (95% CI 66–84%). Similarly, higher levels of serum ferritin was observed in approximately 70.3% (95% CI 61.1–64.2%) of the patients. Elevation of IL-6 was present in about two-thirds of patients (65.9%, 95% CI 55.2%, 76.5%).

Moreover, among cardiac biomarkers, increased levels of NT-pro BNP, d-dimer, and LDH were found in approximately 40% of patients. The estimated pooled frequencies of elevation of NT-pro BNP, d-dimer, and LDH were equal to 46.5% (95% CI 28.9–64.2%), 41.5% (95% CI 31.0–52.1%), and 41.0% (95% CI 28.8–53.2%), respectively. Elevation of cardiac troponins was observed in approximately one-quarter of patients (25.3%, 95% CI 17.6–33.1%). Additionally, we identified and analyzed the findings of five studies that had investigated the number of patients with increased myoglobin levels. The estimated pooled frequency of elevated myoglobin was at near 19.1% (95% CI 11.6–26.6%). Lastly, we found ten studies reporting the number of patients with elevated CK levels and two studies reporting the number of patients with elevated CK-MB levels. The estimated pooled frequency of elevation of CK and CK-MB were equaled to 15.9% (95% CI 10.5–21.3%) and 66.2% (95% CI 6.9–125.6%), respectively. Of note, Chen et al. reported reduction of CK levels in 23% of their patients [18]. As the wide CI range and high heterogeneity score (*I*^2^ = 99%) show, the results of this analysis cannot be very reliable and additional original investigations are required in this regard. Notably, there was significant heterogeneity in the estimates of the frequency of increased cardiac and inflammatory biomarkers (86 %  ≤ *I*^2^ ≤ 99%).

Only two studies had measured the levels of TNF-α. Pooled frequency of elevated levels of TNF-α was 58.3% (95% CI 53.8–62.7%) [19, 33].

##### Association of newly developed acute cardiac injury, pre-existing cardiovascular diseases, hypertension, and diabetes with disease severity and survival

**Fig. 3:**
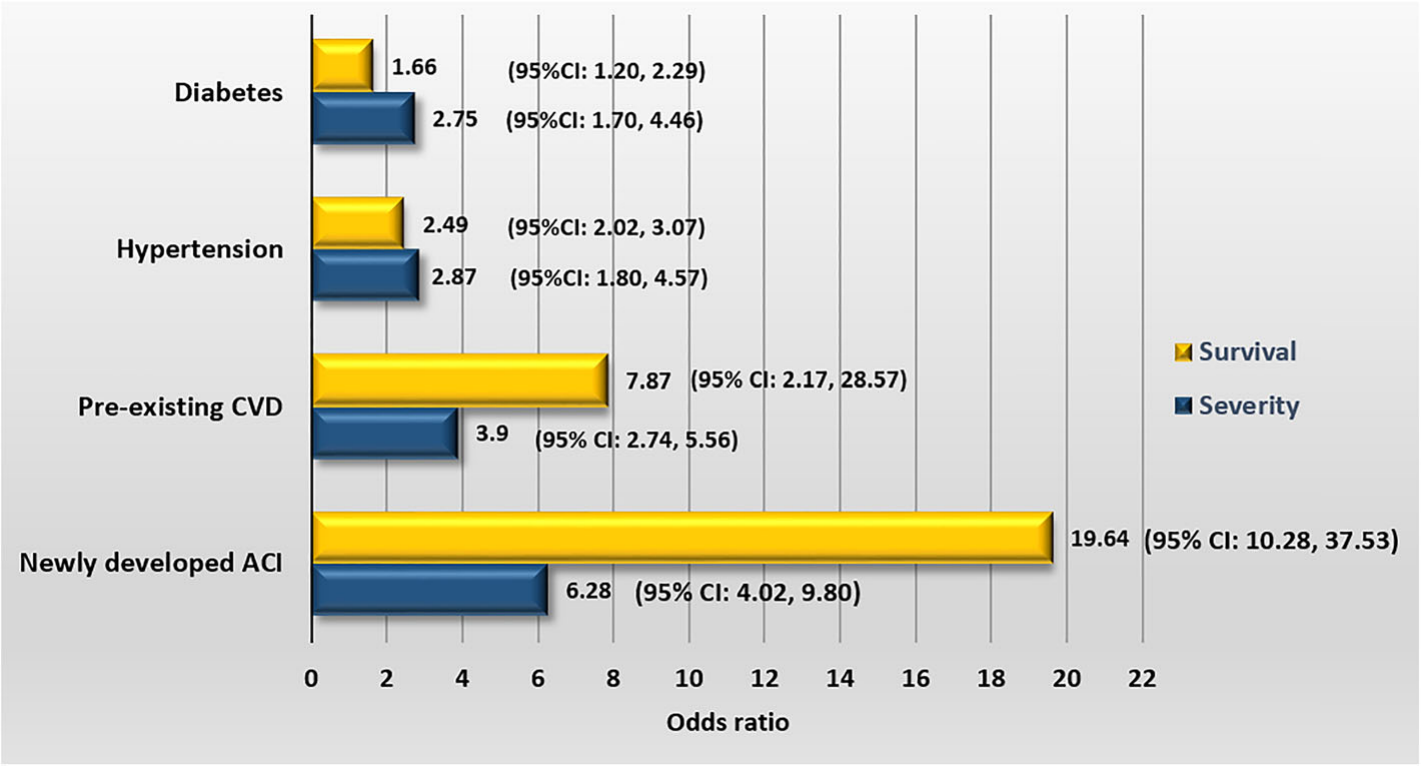
The odds ratio for death and developing severe forms of COVID-19 infection according to the newly developed acute cardiac injury, hypertension, diabetes mellitus, and pre-existing cardiovascular diseases (*ACI* acute cardiac injury, *CVD* cardiovascular disease, *CI* confidence interval)

Figure 3 depicts the odds ratio for death and developing severe forms of COVID-19 infection according to the presence of newly developed acute cardiac injury, hypertension, diabetes mellitus, and pre-existing cardiovascular diseases. The forest plots showing the odds ratio for death according to newly developed acute cardiac injury, pre-existing cardiovascular disease, hypertension, and diabetes mellitus are available in Supplementary Material 6 (S6).

##### Newly developed acute cardiac injury: association with survival and severity

**Fig. 4:**
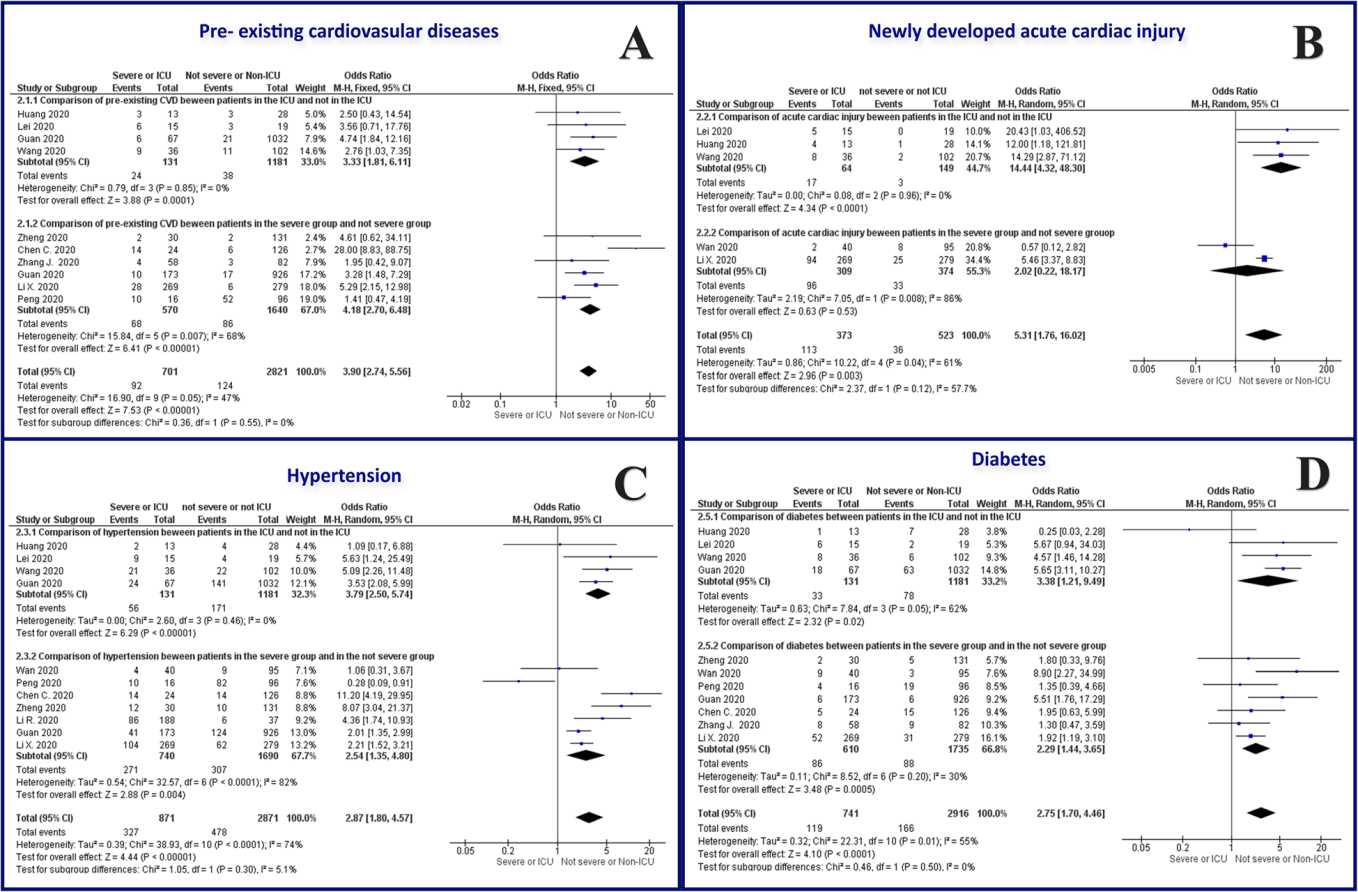
Forest plots showing the odds ratio for severity according to the newly developed acute cardiac injury, pre-existing cardiovascular disease, hypertension and diabetes mellitus

The development of acute cardiac injury increased the risk of mortality by near 20 times (OR 19.64, 95% CI 10.28–37.53, *P <* 0.001). Patients developing acute cardiac injury had a much higher risk of being admitted to ICU (OR 13.5, 95% CI 3.61–50.52, *P* < 0.001). Studies included in the analysis of assessing the effect of acute cardiac injury on the risk of being admitted to ICU were homogenous (*I*^2^ = 0%). When we added one study comparing the incidence of acute cardiac injury between severe and non-severe patients, we found that development of acute cardiac injury increased the occurrence of more severe presentation of the disease more than six times (OR 6.28, 95% CI 4.22–9.8, *P* < 0.001) (Fig. 4).

##### Pre-existing cardiovascular diseases: association with survival and severity

Pre-existing cardiovascular diseases pose a significant risk of mortality. Patients with these conditions were near eight times more likely to have a fatal outcome (OR 7.87, 95% CI 2.17–28.57, *P* = 0.002). Studies comparing pre-existing cardiovascular diseases between deceased and survived patients were moderately heterogeneous (*I*^2^ = 54%,  *P* = 0.07). Similarly, patients with pre-existing cardiovascular diseases were about four times more likely to be categorized in the ICU or severe groups (OR 3.9, 95% CI 2.74–5.56, *P* < 0.001). Patients with pre-existing cardiovascular diseases were approximately three times more likely to be admitted to the ICU (OR 3.33, 95% CI 1.81–6.11, *P* < 0.001) and four times more likely to develop severe forms of the disease (OR 4.18, 95% CI 2.7–6.48, *P* < 0.001) (Fig. 4).

##### Hypertension: association with survival and severity

We included eight studies with low heterogeneity (*I*^2^ = 25%) to assess the role of hypertension in increasing the mortality rate and found that patients with hypertension were more than twice more likely to die from COVID-19 compared to other patients (OR 2.49, 95% CI 2.02–3.07, *P* < 0.001).

Likewise, patients with hypertension were about three times more likely to be categorized in the ICU or severe groups (OR 2.87, 95% CI 1.80–4.57, *P* < 0.001). Patients with hypertension were approximately 3.8 times more likely to be admitted to the ICU (OR 3.79, 95% CI 2.50–5.74, *P* < 0.001) and 2.5 times more likely to develop severe forms of COVID-19 infection (OR 2.54, 95% CI 1.35, 4.80, *P* < 0.001) (Fig. 4).

##### Diabetes: association with survival and severity

Six studies with low heterogeneity (*I*^2^ = 0%) had reported number of patients with diabetes in deceased and survived groups. We found that patients with diabetes were slightly more likely to die from COVID-19 compared to other patients (OR 1.66, 95% CI 1.20–2.29, *P* < 0.001).

Similarly, patients with diabetes were near three times more likely to be categorized in the ICU or severe groups (OR 2.75, 95% CI 1.70–4.46, *P* < 0.001). They were approximately 3.38 times more likely to be admitted to the ICU (95% CI 1.21–9.49, *P* = 0.02) and 2.29 times more likely to develop severe forms of COVID-19 infection (95% CI 1.44, 3.65, *P* < 0.001) (Fig. 4).

##### Elevated levels of cardiac and inflammatory biomarkers level: association with survival

**Fig. 5:**
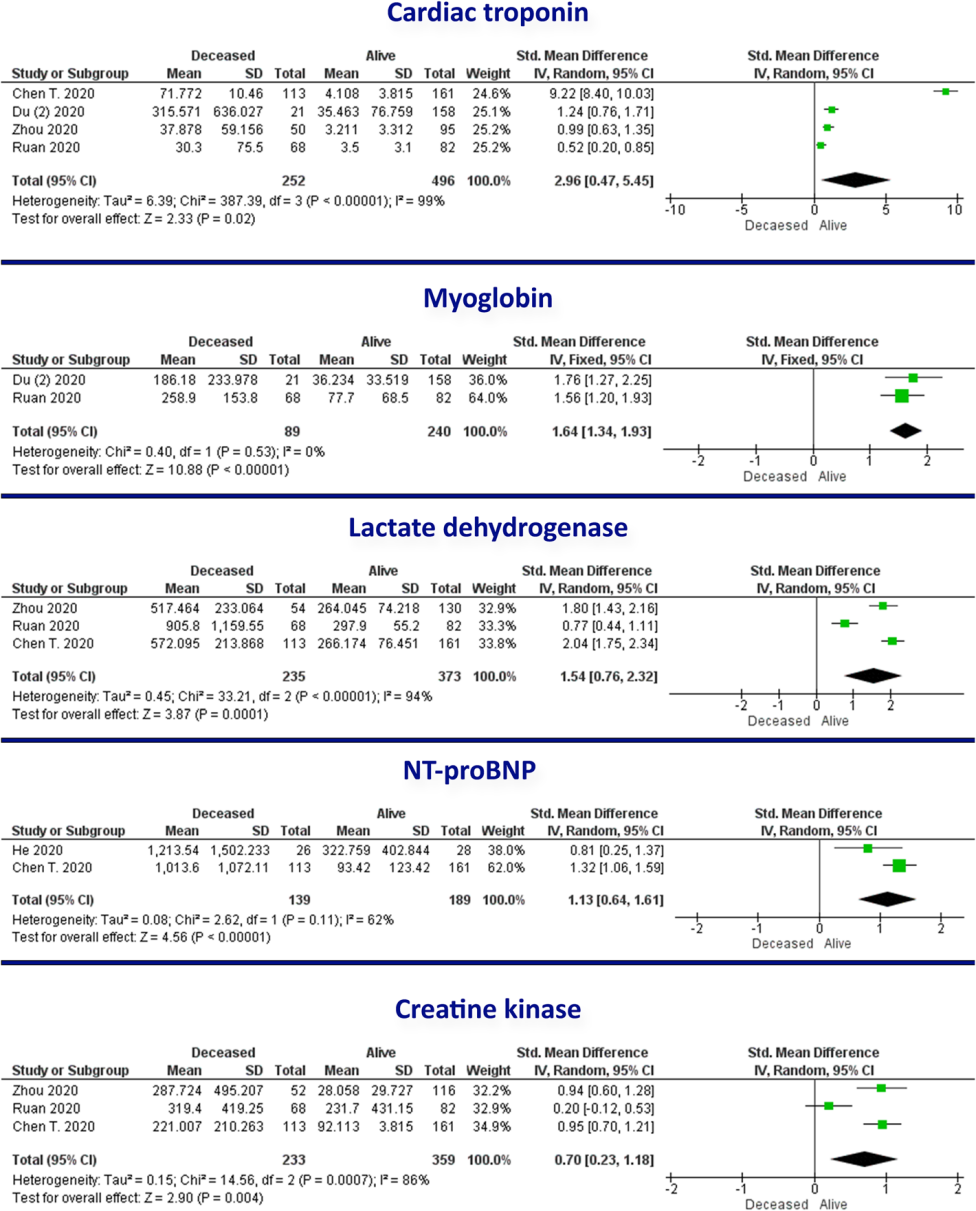
Forest plots showing the standardized mean difference of cardiac biomarkers (troponin, myoglobin, LDH, NT-proBNP and CK) in the severe or ICU group compared with patients in the non-severe or non-ICU group

The levels of cardiac biomarkers, including cardiac troponin (SMD = 2.96, 95% CI 0.47–5.45, *P =* 0.02), myoglobin (SMD = 1.64, 95% CI 1.34–1.93, *P* < 0.001), LDH (SMD = 1.54, 95% CI 0.76–2.32, *P* < 0.001), NT-pro BNP (SMD = 1.13, 95% CI 0.64–1.61, *P* < 0.001), and CK (SMD = 1.13, 95% CI 0.64–1.61, *P* < 0.001) were significantly higher in the deceased group (Fig. 5).

We included elevated levels of troponin I, T, and high-sensitivity troponin (either I or T) as cardiac troponins.

**Fig. 6:**
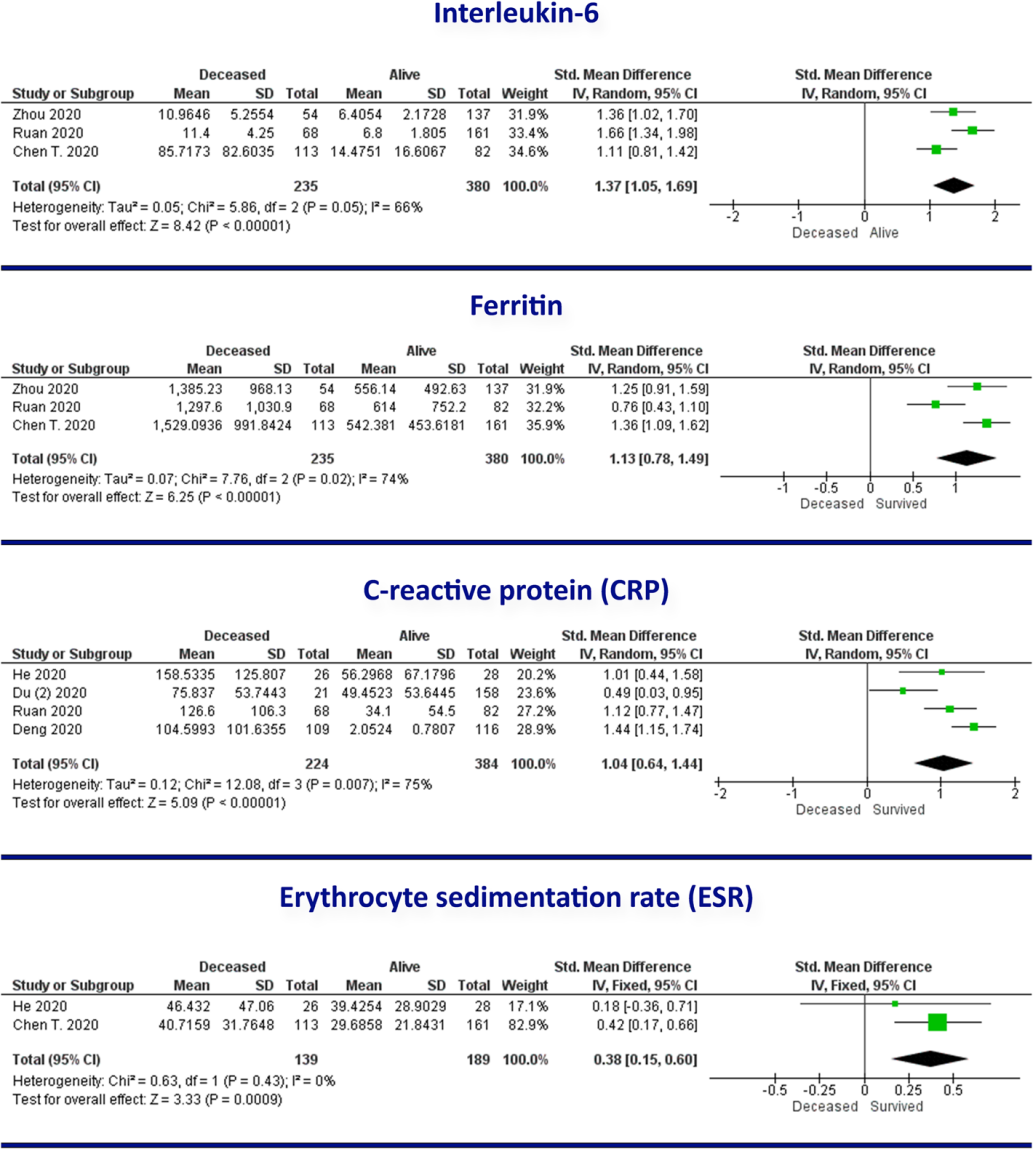
Forest plots showing the standardized mean difference of interleukin-6, ferritin, CRP and ESR between the deceased and survived patients

Similarly, the levels of inflammatory markers, including IL-6 (SMD = 1.37, 95% CI 1.05–1.69, *P* < 0.001), ferritin (SMD = 1.13, 95% CI 0.78–1.49, *P* < 0.001), CRP (SMD = 1.04, 95% CI 0.64–1.44, *P* < 0.001), and ESR (SMD = 0.38, 95% CI 0.15–0.60, *P* < 0.001), were significantly higher in the deceased group (Fig. 6).

##### Cardiac biomarkers levels: association with severity

Overall, LDH levels were higher in patients classified as the ICU or severe groups (SMD = 0.75, 95% CI 0.34–1.16, *P* < 0.001). Notably, the SMD in the severe or non-severe subgroup (SMD = 0.96, 95% CI 0.4–1.52, *P* < 0.001) was larger than the SMD in the ICU or not ICU subgroup (SMD = 0.5, 95% CI 0.05–0.96, *P* = 0.03).

Overall, CK levels were higher in patients classified as the ICU or severe groups (SMD = 0.55, 95% CI 0.27–0.82, *P* < 0.001). Additionally, the SMD in ICU or not ICU subgroup (SMD = 0.58, 95% CI 0.28–0.88, *P* < 0.001) was quite similar to the severe or non-severe subgroup (SMD = 0.54, 95% CI 0.09–0.99, *P* = 0.02).

**Fig. 7:**
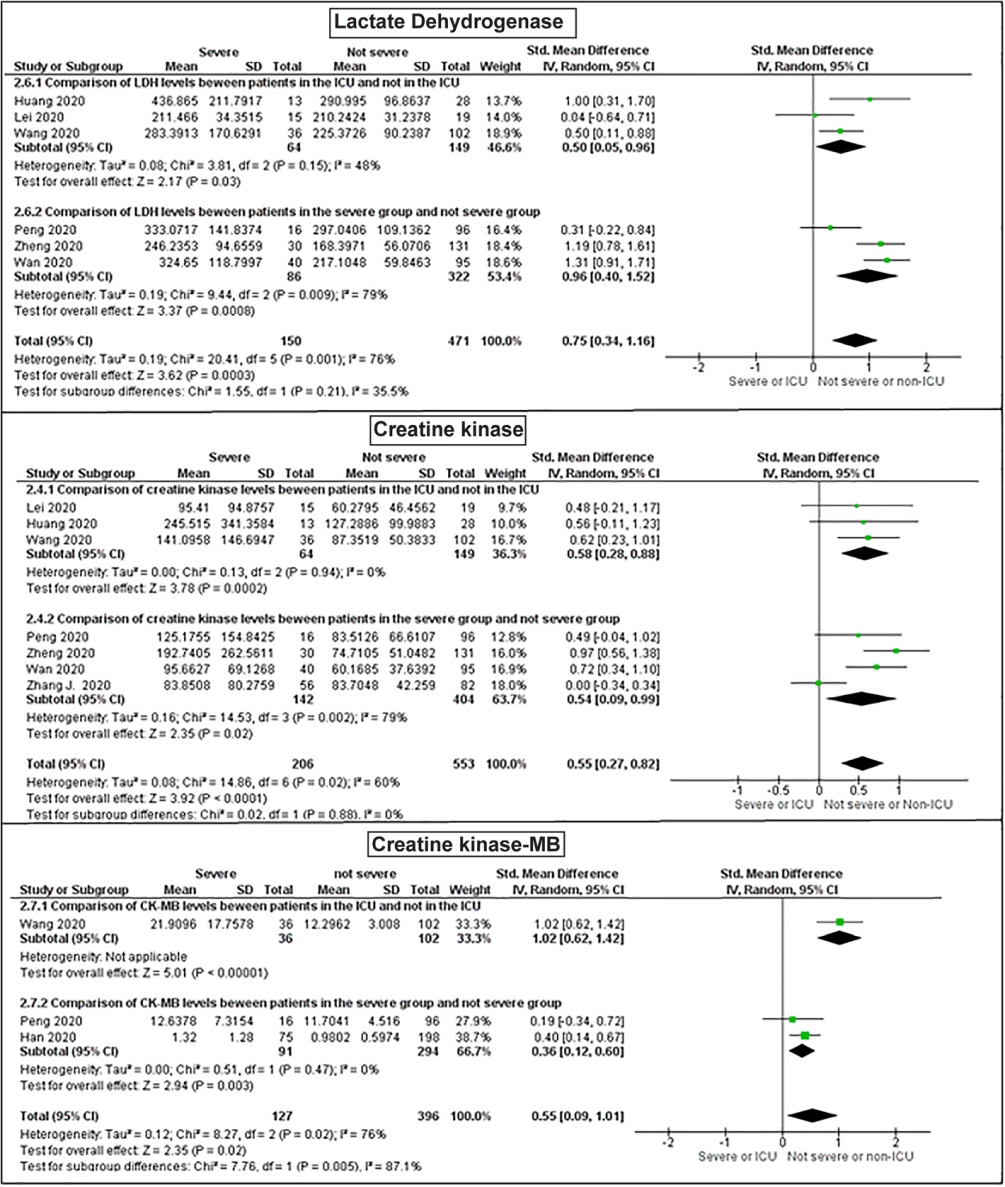
Standard mean difference of LDH, CK and CK-MB between COVID-19 patients with severe disease in comparison with non-severe disease

Two studies had assessed the difference between CK-MB levels between the severe and non-severe group of the patients. CK-MB levels were higher in the patients with severe forms of COVID-19 (SMD = 0.36, 95% CI 0.12–0.6, *P* < 0.001). When we added the only study which had compared the levels of CK-MB between ICU and non-ICU patients, the total SMD was 0.55 (95% CI 0.09–1.01, *P* = 0.02) (Fig. 7).

##### Pooled frequencies of acute cardiac injury, acute hepatic injury, and acute kidney injury

Given the cardio-hepatic and cardio-renal interactions, we also analyzed the pooled frequency of acute liver injury and acute kidney injury in our selected studies [48–52].

While acute cardiac injury was reported in 25.3% (95% CI 19.5–31.1%) of patients, acute liver injury was reported in 22.2% (95% CI 13.5–31.0%) and acute kidney injury was stated in 8.8% of studies (95% CI 5.4–12.2%).

Funnel plots showing publication bias of studies are shown in Supplementary Material 7 (S7).

### Systematic review

#### Spectrum of newly developed cardiovascular diseases and the mechanisms of acute cardiac injury (adult versus pediatric population)

A diverse range of cardiovascular disease has been reported in patients with COVID-19 which encompasses acute myocarditis (including acute lymphocytic myocarditis) [53], fulminant myocarditis [15, 24, 37, 54–56], acute myocardial infarction type 2 [7], acute myopericarditis [46], acute pulmonary embolism [57–60], cardiac tamponade [46], cardiogenic shock [61, 62], cardiomyopathy [63], heart failure, pericardial effusion, pulmonary hypertension, reverse Takotsubo cardiomyopathy [64], and right ventricular dysfunction [62, 65].

Left ventricular failure was more commonly reported in COVID-19 patients than right ventricular dysfunction. However, to date, the majority of cases with right ventricular failure were secondary to acute pulmonary embolism [65, 66]. Nevertheless, Fried and his colleagues reported myopericarditis in a 64-year-old patient who developed biventricular failure and was successfully managed using an intra-aortic balloon pump (IABP) [62].

In the pediatric population, there was a case of mild elevation of troponin I in a 55-day-infant with excellent outcome and discharge from the hospital [67]. However, both Kawasaki-like disease and Kawasaki shock-like syndrome have been reported with COVID-19. Jones et al. reported a 6-month-old female infant with the typical symptoms of Kawasaki disease that was tested positive for COVID-19. Her echocardiogram was normal [68]. Riphagen and her colleagues reported 8 patients with shock, aged 4 to 14 years, who had initially presented with persistent fever, rash, inflammation of the conjunctiva, peripheral edema, and pain in the extremities. All except one had prominent gastrointestinal symptoms, and all except one weighed above 75th percentile. All had elevated levels of cardiac troponins and developed warm shock. None of them tested positive for COVID-19. However, a 14-year-old boy with 95 kg weight and a BMI of 33kg/m^2^, who underwent ECMO and died of cerebral infarction was tested positive postmortem, and another case was positive for COVID-19 after discharge. Antibody test was positive for COVID-19 in all the eight patients. Their echocardiographic findings included left ventricular and/or right ventricular dysfunction and increased brightness of walls of the coronary arteries. The authors suggested that COVID-19 can produce a “hyper-inflammation syndrome” that involves multiple organs, mimicking Kawasaki disease shock syndrome [69].

The exact mechanisms of cardiac injury in patients with COVID-19 are not confirmed. However, it is speculated that cardiac injury can occur through one or more of the putative mechanisms [28, 70, 71]: direct invasion by the virus, indirect damage due to the systemic inflammatory syndrome and cytokine storm, dysregulation of renin-angiotensin-aldosterone system, hypoxia-induced cardiac injury, microvasculature damage of the heart, stress-induced cardiomyopathy, and cardiac damage secondary to multi-organ failure.

Timing of the appearance of the cardiac complications, early versus late stage, may serve as a hint to the diagnosis of the putative mechanism/s [72, 73].

#### Spectrum of newly developed arrhythmias and electrocardiographic changes

Dynamic changes in ECG are of paramount importance during COVID-19 and imply acute cardiac derangement. Patients with COVID-19 are at risk of arrhythmias and ECG changes due to the disease itself or because of the medications used for its prevention or treatment, such as chloroquine, hydroxychloroquine, and azithromycin. These three medications can prolong QT interval and predispose the patient to torsades de pointes [74]. Nevertheless, to date, no case of torsades de pointes due to QTc prolongation secondary to consumption of these drugs has been reported in these patients.

Sinus tachycardia is the most common rhythm disturbance reported in patients with COVID-19. Moreover, a variety of other rhythm disorders and electrocardiographic alterations have been reported in patients with COVID-19 such as supraventricular tachycardia [62, 63], ventricular tachycardia, first-degree atrioventricular block (AVB), temporary second-degree AVB, reversible complete heart block, generalized ST-elevation masquerading ST-elevation myocardial infarction (STEMI), triangular-type ST-elevation myocardial infarction type 2, S1Q3T3 pattern mimicking acute pulmonary embolism, non-specific ST and T wave changes, diffuse U waves in the presence of a QTc of 0.45 ms, and pulseless electrical activity [63, 75].

#### Spectrum of pathologic findings on cardiac biopsy or autopsy

By 23rd of April 2020, there are seven studies reporting the cardiac pathological findings [7, 61, 64, 76–79]. The summary of these studies is tabulated in supplementary Table 3 in Supplementary Material 8 (S8). Two of these have also performed electron microscopic examination [61, 79]. Only in one case with fulminant myocarditis and cardiogenic shock, virus was present in the pericytes [61]. No obstruction or thrombosis of epicardial coronary arteries were reported. Prominent infiltration of T lymphocytes was present in only one case which presented as reverse Takutsubo cardiomyopathy [53].

## Discussion

This study showed that compared to the liver and kidney, the heart was the second most commonly involved organ affected by COVID-19 after the lungs. Slightly more than one-fourth of the hospitalized patients with COVID-19 develop CVD and arrhythmia, which increased the mortality by nearly 20 times and the need for ICU admission by 13.5 times. Hypertension was present in slightly less than one-third of admitted patients and led to increased mortality and the need for ICU admission by approximately 2.5- and 4-folds, respectively. However, this association may be partly due to the fact that the incidence of hypertension increases with aging [80]. It is already known that mortality is higher in older patients with COVID-19 [81]. The relationship among inflammation, oxidative stress, and vascular dysfunction, known as vascular health triad, is reported as a common mechanism between aging and hypertension [82].

Patients with pre-existing heart failure had 8-fold more death and nearly 3.5-fold more need for the ICU admission.

In a systematic review and meta-analysis, Santoso et al. studied 2389 patients, and by calculating the risk ratio showed that in patients with cardiac injury, mortality and need for ICU admission were 7.95 times and 7.94-fold more likely, respectively [83].

Not only do cardiovascular complications increase morbidity and mortality, but they also enhance the risk of sepsis and septic shock. This may be explained by the prolonged period of hospitalization and/or use of different invasive devices to support the circulatory failure [56, 61].

Even after discharge, patients with a history of myocarditis may develop myocardial scars, which predispose them to cardiac arrhythmia. Follow-up investigation by cardiac magnetic resonance imaging is recommended in order to determine the risk of cardiac arrhythmias [84]. Another reason for the necessity of follow-up is to ensure that acute myocarditis of COVID-19 does not evolve into dilated cardiomyopathy in later life [85]. Furthermore, cardiac magnetic resonance imaging can be of great help in the diagnosis of acute myocarditis in hospitalized patients; nevertheless, it was used scarcely [86].

Pulmonary embolism was frequently reported and should be considered in any patient with COVID-19 who experience sudden deterioration of clinical condition associated with an acute drop in oxygen saturation or those with significantly elevated levels of d-dimer.

COVID-19-associated coagulopathy (CAC), the etiology of which is multifactorial and not yet completely understood, has been reported with a spectrum of manifestations ranging from hypercoagulability (in the vast majority of the reported cases) to less common reports of bleeding, particularly in patients treated with ECMO or anticoagulants [87]. The triad of Virchow, including endothelial damage, alterations in blood flow and presence of prothrombotic components in the circulation, appears to have a substantial role in the development of CAC [87].

The spectrum of presentation of hypercoagulability states in COVID-19 encompasses a wide range that spans from localized microvascular thrombosis in the lungs or pulmonary intravascular coagulopathy (PIC) to systemic venous and arterial thrombosis, including aortic thrombosis [88–90]. Development of COVID-19-associated coagulopathy is associated with a worse prognosis [91]. Hence, prophylactic treatment with low molecular weight heparin is recommended for all hospitalized COVID-19 patients, unless there is a contraindication [92, 93]. Anticoagulant therapy has been shown to be associated with improved survival [94].

Our meta-analyses demonstrated that IL-6, which has a critical role in cytokine release syndrome, was elevated in approximately two-thirds of the hospitalized patients. Coomes and his colleagues, in a recent systematic review and meta-analysis on the role of IL-6 in patients with COVID-19, showed that IL-6 was 2.9 times higher in patients with complicated COVID-19, compared with the non-complicated group. They also found that IL-6 levels were 3.24-fold higher in the 33 patients who required ICU admission in comparison with 507 patients who did not need to be admitted in the ICU [95]. Increased IL-6 can also explain the speculative cardiac microvascular abnormalities in patients with COVID-19 [27]. Zhang et al. highlighting the substantial role of IL-6, described the pathophysiology of cytokine release syndrome in patients with COVID-19 and severe clinical course. They proposed that tocilizumab, a recombinant humanized monoclonal antibody against the receptor of IL-6, can serve as a potentially effective treatment for cases presenting as severe COVID-19 [96]. Given the central role of IL-6 in cytokine release syndrome and the safety of tocilizumab in children and adult patients, Liu et al. also proposed tocilizumab as a treatment for patients with severe COVID-19 [97].

We observed higher levels of cardiac troponin, myoglobin, LDH, NT-proBNP, CK, and IL-6, in the deceased patients in comparison with the survived. Li and his colleagues showed that cardiac troponins and NT-proBNP were higher in the deceased patients with COVID-19. They also reported that the dynamic rise in the latter biomarkers was only observed in the deceased group [15]. Likewise, Guo et al. studied 187 patients with COVID-19 and observed that dynamic changes or rising levels of NT-proBNP and cardiac troponin T were significantly higher in the deceased patients [98].

We found a sharp contrast between frequency, severity, and outcome of cardiac involvement due to COVID-19 in pediatric versus adult population [99].

In a systematic review of children with COVID-19, Ludvigsson et al. did not report even one case of COVID-19-induced cardiac injury in the pediatric age range. They cited the study of Dong et al. as the largest study on children, the study in which only 3 cases of the 724 confirmed cases had the critical disease, none of them had cardiac injury [100, 101]. Shekerdemian et al. reported 48 children with COVID-19 who were admitted to ICU and 83% of whom had significant underlying comorbidities, including congenital heart disease and cardiomyopathy in three cases. Although they reported a fatality rate of 4%, they have not delineated whether the deceased patients were those with prior cardiac disease. Furthermore, except the one patient with underlying cardiomyopathy who underwent veno-arterial ECMO for treatment of cardiogenic shock, no further information regarding acute cardiac injury is provided [102].

Given the fact that angiotensin-converting enzyme 2 (ACE2) functions as the receptor for the entry of SARS-CoV-2 into the cell, the answer may be partly hidden in the age-related differences in the renin-angiotensin-aldosterone system (RAAS) [103]. This hypothesis is supported by the evidence that shows the elderly are predisposed to severe forms of the COVID-19 [104–106]. Furthermore, Musso et al. reported that advancing age adversely affects the RAAS [107]. Similarly, senescence-related changes in the immune function may also be contributing [108].

Nevertheless, it seems that although mortality due to direct viral invasion to the heart is not reported in children, the catastrophic effects of hyper-inflammatory response with multi-organ involvement including the heart, the so-called COVID-19 pediatric hyper-inflammatory shock syndrome, can be life-threatening. In fact, both Kawasaki-like disease and Kawasaki shock-like syndrome have been reported with COVID-19. These findings may also potentially shed light on the role of viruses in the pathogenesis of Kawasaki disease.

### Triage risk stratification tool for patients with COVID-19 and cardiovascular disease

Time of presentation of the cardiac disorders (early stage versus late stage) and levels of various cardiac and inflammatory biomarkers can serve as clues to the putative underlying mechanism/s. To detect patients at the highest risk at the earliest, i.e., at triage, a simple and practical plan is necessary to guide the clinician on the minimal essential initial laboratory work-up as a guide for pathophysiologically targeted treatment.

**Fig. 8:**
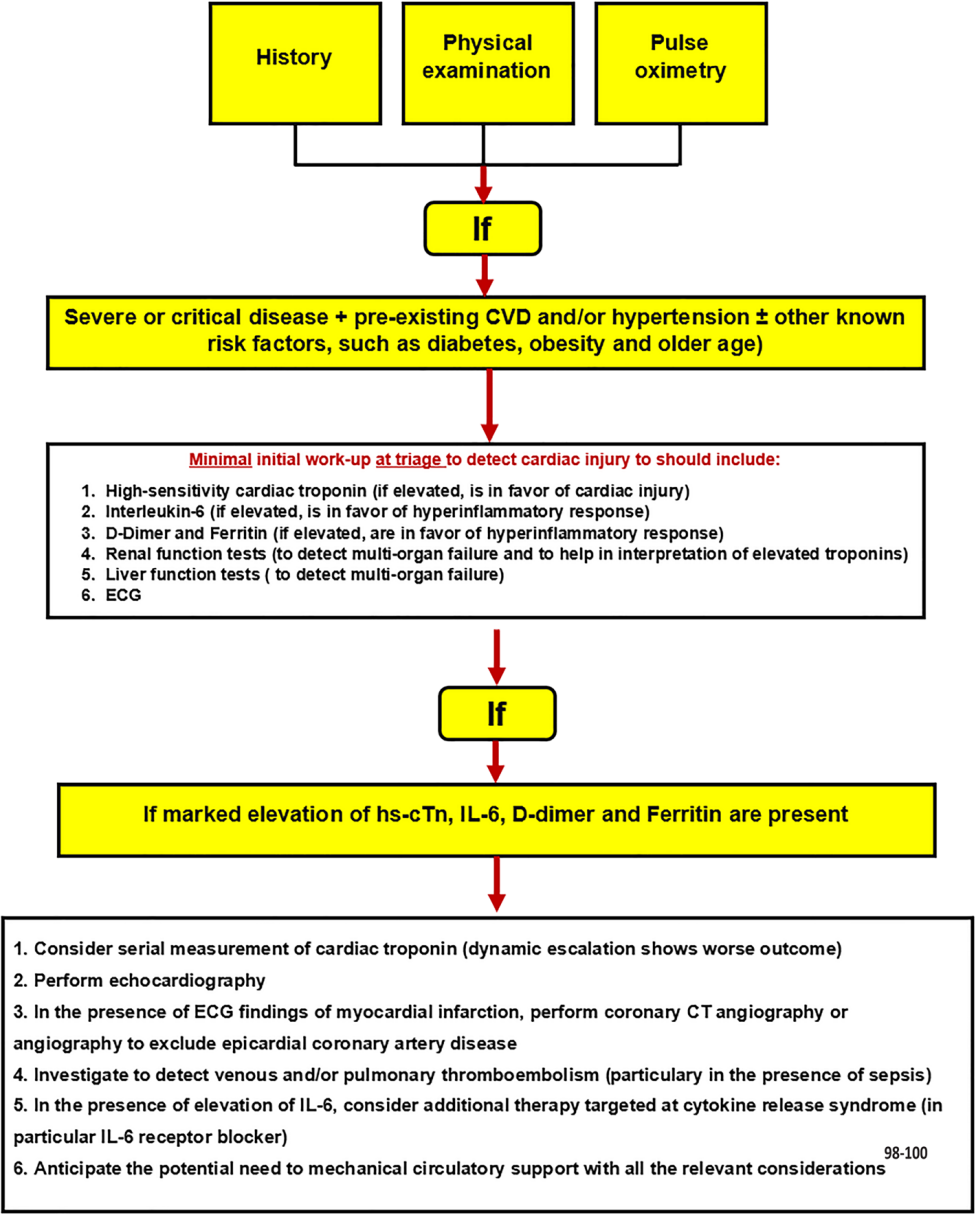
Triage risk stratification tool for high-risk patients with COVID-19 and cardiovascular disease

Figure 8 shows a proposed triage risk stratification tool for patients with COVID-19 and CVD.

Two considerations should be taken into account when using this tool. The first is that the laboratory investigations in this tool are in addition to the routine laboratory evaluations such as CBC, ESR, CRP, and blood glucose. Secondly, it should be borne in mind that for consideration of mechanical circulatory support, the conventional indications and contraindications in patients with COVID-19, in addition to the precautions for prevention and treatment of infections, should be strictly followed [109–111].

## Conclusion

This study showed that pre-existing and newly developed cardiovascular disease are common in patients with COVID-19 and are associated with increased severity and mortality in these patients. The frequency, clinical pattern, severity, and outcome of cardiovascular injury in pediatric patients with COVID-19 is not only different from the adult population but also varies in the pediatric age range, ranging from no cardiac damage in the majority of cases to Kawasaki Shock-like syndrome in the minority. We proposed a triage risk stratification tool in patients with COVID-19 and CVD for timely recognition of the high-risk patients and well-timed establishment of the pathophysiologically targeted treatment. This tool needs to be validated in future studies.

Lastly, COVID-19 should be regarded as a disease that can affect multiple organs with multiple mechanisms, each of which may need therapy targeted at the mechanism of injury in order to be effective.

## Limitations

There were significant inconsistencies in reporting cardiovascular derangements in patients with COVID-19. Consistent terminology and definition of cardiac injury were not used in the studies. Furthermore, the *I*^2^ index was high in some meta-analyses. Certain studies had done intensive evaluation and had presented immense information, whereas there was a dearth of information regarding the patients in others. These limitations indicate the necessity of a standardized terminology to report cardiovascular complications and a standard diagnostic approach to patients with COVID-19 worldwide. We did not include cardiac arrest in our analysis because the precise underlying pathophysiology of dying process (end-stage respiratory failure, cardiogenic shock, multiple organ failure, or cardiogenic shock) was not delineated in the majority of studies [112]. We could not include obesity in our study because data in this regard was not provided in the majority of studies. Another limitation of this study was that we did not search Cochrane, CINAHL, Web of Science, Scopus, and Google Scholar database in our online search [113]. Lastly, the limitations due to the possible inherent biases in the non-randomized cohort data apply to this study.

## Supplementary information

**Additional file 1: Supplementary Material 1 (S1)** Search strategy

**Additional file 2: Supplementary Material 2 (S2) Supplementary Table 1.** Summary of the 35 studies included in the meta-analysis

**Additional file 3: Supplementary Material 3 (S3) Supplementary Table2.** Inflammatory and cardiac biomarkers in COVID-19

**Additional file 4: Supplementary Material 4 (S4)** Risk of bias assessment

**Additional file 5: Supplementary Material 5 (S5) Figure 1. ** Forest plot of the pooled frequency analyses ( including Figure 1. Forest plot of the pooled frequency analysis of newly developed acute cardiac injury. **Figure 2.** Forest plot of the pooled frequency analysis of newly developed arrhythmia. **Figure 3.** Forest plot of the pooled frequency analysis of newly developed heart failure. **Figure 4.** Forest plot of the pooled frequency analysis of chest pain or chest tightness. **Figure 5.** Forest plot of the pooled frequency analysis of palpitation. **Figure 6.** Forest plot of the pooled frequency analysis of hypertension. **Figure 7.** Forest plot of the pooled frequency analysis of pre-existing cardiovascular diseases. **Figure 8.** Forest plot of the pooled frequency analysis of pre-existing heart failure. **Figure 9.** Forest plot of the pooled frequency analysis of pre-existing diabetes. **Figure 10.** Forest plot of the pooled frequency analysis of patients with elevated NT-pro BNP levels. **Figure 11.** Forest plot of the pooled frequency analysis of patients with elevated cardiac troponins levels. **Figure 12.** Forest plot of the pooled frequency analysis of patients with elevated creatine kinase-MB levels. **Figure 13.** Forest plot of the pooled frequency analysis of patients with elevated creatine kinase levels. **Figure 14.** Forest plot of the pooled frequency analysis of patients with elevated D-dimer levels. **Figure 15.** Forest plot of the pooled frequency analysis of patients with elevated lactate dehydrogenase levels. **Figure 16.** Forest plot of the pooled frequency analysis of patients with elevated interleukin- 6 levels. **Figure 17.** Forest plot of the pooled frequency analysis of patients with elevated C-Reactive Protein (CRP) levels. **Figure 18.** Forest plot of the pooled frequency analysis of patients with elevated erythrocyte sedimentation rate (ESR) levels. **Figure 19.** Forest plot of the pooled frequency analysis of patients with elevated ferritin levels).

**Additional file 6:** Forest plots showing odds ratio for death according to newly developed acute cardiac injury, pre-existing cardiovascular disease, hypertension and diabetes mellitus.

**Additional file 7: Supplementary Material 7 (S7) Figure 1.** Funnel plot of studies comparing frequency of acute cardiac injury between severe or ICU group and non-severe or non-ICU group showing publication bias. **Figure 2.** Funnel plot of studies comparing frequency of pre-existing cardiovascular diseases between severe or ICU group and non-sever or non-ICU group showing publication bias. **Figure 3.** Funnel plot of studies comparing frequency of pre-existing cardiovascular disease between severe or ICU group and non-severe or non-ICU group showing publication bias. **Figure 4.** Funnel plot of studies comparing frequency of acute cardiac injury between deceased and survived patients showing publication bias

**Additional file 8:** Supplementary Material 8 (S8) Supplementary Table 3*—*Cardiac pathologic findings in 13 deceased patients with COVID-19*.* A: absent, CAA: coronary artery atherosclerosis, CAD: coronary artery disease, ECG: electrocardiography, EM: electron microscopy, hs-cTn: high-sensitivity cardiac troponin I, LVEF: left ventricular ejection fraction, NS: not stated, P: present

## Data Availability

Data is available on request.

## Abbreviations

ACE2: Angiotensin-converting enzyme 2
AVB: Atrioventricular block
CAC: COVID-19-associated coagulopathy
CBC: Complete blood count
CI: Confidence interval
CK: Creatine kinase
CK-MB: Craetine-kinase-MB
COVID-19: Coronavirus disease 2019
CRP: C-reactive protein
CVD: Cardiovascular disease
ECMO: Extracorporeal membrane oxygenation
ESR: Erythrocye sedimentation rate
IABP: Intraaortic balloon pump
ICU: Intensive care unit
IL-6: Interleukin-6
IQR: Interquartile range
LDH: Lactate dehydrogenase
NT-proBNP: N-terminal-pro B-type natriuretic peptide
OR: Odds ratio
PRISMA: Preferred Reporting Items for Systematic Reviews and Meta-Analyses
RAAS: Renin-angiotensin-aldosterone system
S: Supplementary
SD: Standard deviation
SMD: Standard mean difference
Tn: Troponin
TNF-α: Tumor necrosis factor-alpha
TRST: Triage risk stratification tool
