# Supplementary material for: Cardiovascular disease in COVID-19: a systematic review and meta-analysis of 10,898 patients and proposal of a triage risk stratification tool
Source: Egypt Heart J. 2020 Jul 13;72:41. doi: 10.1186/s43044-020-00075-z (PMC7356124; doi:10.1186/s43044-020-00075-z)
Supplement: Supplementary file 1 — Additional file 1: Supplementary Material 1 (S1) Search strategy [file 43044_2020_75_MOESM1_ESM.docx]

**Supplementary Material 1 (S1)**

**Search Strategy**

**The search strategy in PubMed:**

heart[Title/Abstract] OR "cardiac magnetic resonance imaging"[Title/Abstract]) OR echocardiography[Title/Abstract]) OR cardiac[Title/Abstract] OR electrocardiography[Title/Abstract]) OR hypertension[Title/Abstract] OR arrhythmia[Title/Abstract] OR "myocardial injury"[Title/Abstract] OR "coronary artery"[Title/Abstract]) OR "pericardial effusion"[Title/Abstract] OR "pulmonary embolism"[Title/Abstract] OR angiography[Title/Abstract] OR "Cardiovascular Diseases"[Mesh] OR "Cardiomyopathies"[Mesh] OR "Heart Failure"[Mesh] OR "Endocarditis"[Mesh] OR "Hypertension"[Mesh] OR "Shock"[Mesh] OR "Myocarditis"[Mesh]) OR "Pericarditis"[Mesh] OR tamponade[Title/Abstract] OR hypotension[Title/Abstract] OR pericarditis[Title/Abstract] OR myocarditis[Title/Abstract]

AND

"COVID-19" [Supplementary Concept] OR “2019 novel coronavirus disease” OR “COVID19” OR “coronavirus disease-19” OR “2019 novel coronavirus infection” OR “2019-nCoV”

**The search strategy in Embase:**

heart:ab,ti OR 'heart muscle injury':ab,ti OR 'heart injury':ab,ti OR 'lung embolism':ab,ti OR hypertension:ab,ti OR shock:ab,ti OR 'coronary artery disease':ab,ti OR 'heart tamponade':ab,ti OR 'heart arrhythmia':ab,ti OR 'pericardial effusion':ab,ti OR electrocardiography:ab,ti OR cardiac:ab,ti OR echocardiography:ab,ti OR angiography:ab,ti OR 'cardiovascular magnetic resonance':ab,ti OR pericarditis:ab,ti OR myocarditis:ab,ti OR cardiomyopathy:ab,ti OR 'heart failure':ab,ti

AND

'covid 19':ab,ti OR '2019 ncov':ab,ti OR covid19:ab,ti OR '2019 novel coronavirus':ab,ti
