# Supplementary material for: Cardiovascular disease in COVID-19: a systematic review and meta-analysis of 10,898 patients and proposal of a triage risk stratification tool
Source: Egypt Heart J. 2020 Jul 13;72:41. doi: 10.1186/s43044-020-00075-z (PMC7356124; doi:10.1186/s43044-020-00075-z)
Supplement: Supplementary file 2 — Additional file 2: Supplementary Material 2 (S2) Supplementary Table 1. Summary of the 35 studies included in the meta-analysis [file 43044_2020_75_MOESM2_ESM.docx]

**Supplementary Material 2 (S2)**

**Supplementary Table 1. Summary of the 35 studies included in the meta-analysis**

| No. | Author | Journal | Study design | No. of patients | Male  N (%) | Age  [Mean (SD) or median (IQR)] | Previous CVD | Evolving CVD | Acute Kidney injury | Elevated  D-dimer | outcome |
| --- | --- | --- | --- | --- | --- | --- | --- | --- | --- | --- | --- |
| 1 | Arentz M. 2020 | JAMA | Case series | 21  Total | 52% | 70 (43 -92) | 9 (42.9%) CHF | 7 (33.3%) Cardiomyopathy | 4 (19.1%) | N/A | 11 (52.4%) Deceased  2 (9.5%) Survived to transfer out of ICU  8 (38.1%) Remains critically ill and requires mechanical  ventilation |
| 2 | Chen N. 2020 | Lancet | Retrospective cohort | 99  Total | 67.7% | 55.5 (13.1) | 40 (40%) CVD | 1 (1%) Heart failure (did not survive) | 3 (3%) | 36 (36%) | 11 (11%) Deceased  31 (31%) Survived  57 (58%) remained hospitalized |
| 3 | Du Y. 2020 | American journal of respiratory and critical care medicine | Retrospective cohort | 85  Total | 77.9% | 65.8 (14.2) | 10 (11.8%) CAD  32 (37.6%) HTN | 51 (60%) Arrhythmia  69 (81.2%) Shock  2 (2.4%) Chest pain (at onset)  **Among causes of death:**  4/81(4.94%) ACS  2/81 (2.47%) Malignant arrhythmia  7/81 (8.64%) Cardiac arrest  38 (44.7%) ACI | 16 (18.8%) | 53 (62.4%) | Deceased |
| 4 | Garg S. 2020 | Morbidity and Mortality Weekly Report  CDC | population-based surveillance | 178  Total | 49% | N/A | 79 (44.3%) HTN  23 (12.9%) CAD  11 (6.17%) CHF | 27 (15%) Chest pain | N/A | N/A | N/A |
| 5 | Grasselli G. 2020 | JAMA | Retrospective observational study | 1591  Total | 82% | 63 (56-70) | 509 (49%) HTN  223 (21%) CVD | N/A | N/A | N/A | 405 (26%) Deceased in ICU  256 (16%) Discharged from ICU  920 (58%)  Remaining in ICU as of 3/25/2020 |
| 6 | Li X. 2020 | International Journal of Infectious Diseases | Retrospective review | 25  Total | 40% | 73 (55-100) | 16 (24%) HTN  5 (32%) CVD | 11 (73.3%) ACI | N/A | 9/12 (75%) | 25 (100%) Deceased |
| 7 | Liu K. 2020 | Chinese medical journal | Retrospective case series | 137  Total | 44.5% | 57 (20–83) | 10 (7.3%) CAD  13 (9.5%) HTN | N/A | N/A | N/A | 16 (11.7%) Deceased  77 (56.2%) Inpatient treatment |
| 8 | Sun C. 2020 | Chinese Journal of Tuberculosis and Respiratory Diseases | Retrospective analysis | 150  Total | N/A | N/A | 13 (8%) HTN | N/A | N/A | 44 (29%) | 3(2%) Deceased  76 (51%) Remained hospitalized  67 (45%) Discharged  4 (2%) transfer |
| 9 | Xu X. 2020 | European Journal of Nuclear Medicine and Molecular Imaging | Retrospective study | 90  Total | 43% | 50 (18–86) | 17 (19%) HTN  3 (3%) CVD | 1 (1%) Pericardial effusion | N/A | N/A | N/A |
| 10 | Chen T. 2020 | BMJ | Retrospective cohort | 274  Total | 103 (38%) | 62 (44, 70) | 93 (34%) HTN  23 (8%) CVD  1(<1%) Chronic heart failure | 103 (38%) chest tightness (at onset)  43/176 (24%) Heart failure  89/203 (44%) ACI | 29 (11%) | 37/247 (15%) | 161 Survived |
|  |  |  |  | 113  Deceased | 55 (49%) | 68 (62, 77) | 54 (48%) HTN  16(14%) CVD  1(1%) Chronic heart failure | 55 (49%) chest tightness (at onset)  41 of 83 (49%) Heart failure  72 of 94 (77%) ACI | 28 (25%) | 34/97 (35%) | Deceased |
|  |  |  |  | 161 Recovered | 88 (55%) | 51 (37, 66) | 39 (24%) HTN  7(4%) CVD | 48 (30%) chest tightness (at onset)  3 of 94 (3%) Heart failure  18 of 104 (17%) ACI | 1 (1%) | 3 /150 (2%) | Survived |
| 11 | Deng Y. 2020 | Chinese medical journal | Retrospective cohort | 109  Deceased | 73 (67.0%) | 69 (62, 74) | 40 (36.7%) HTN  13 (11.9%) CVD | 65 (59.6%) ACI  13(11.9) Shock  11 (10.1%) palpitation (at onset) | 20 (18.3%) AKI | N/A | Deceased |
|  |  |  |  | 116 Recovered | 51 (44.0%) | 40 (33,57) | 18 (15.5%) HTN | 1 (0.9%) ACI  13(11.2%) palpitation | 0 | N/A | Survived |
| 12 | Du RH.2020 | European respiratory journal | Prospective Cohort Study | 179  Total | 54.2% | 57.6±13.7 | 58 (32.4%) HTN  29 (16.2%) CVD or cerebrovascular  diseases | N/A | N/A | N/A | 21 (11.7%) Deceased |
|  |  |  |  | 21  Deceased | 47.6% | 70.2±7.7 | 13 (61.9%) HTN  12 (57.1%) CVD or cerebrovascular  diseases | 13 (61.5%) ACI |  | 76.2% |  |
|  |  |  |  | 158  Recovered | 55.1% | 56.0±13.5 | 45 (28.5&) HTN  17 (10.8%) CVD or cerebrovascular  diseases | 27 (17.9%) ACI |  | 47.9% |  |
| 13 | Ruan Q. 2020 | Intensive Care Medicine | Retrospective study | 68  Deceased | 72% | 67 (15-81) | 29 (43%) HTN  13 (19%) CVD | N/A | 21 (31%) AKI | N/A | Deceased |
|  |  |  |  | 82  Recovered | 65% | 50 (44-81) | 23 (28%) HTN  0 CVD | N/A | 2 (2%) AKI |  | Survived |
| 14 | Zhou F. 2020 | Lancet | Retrospective cohort | 191  Total | 119 (62%) | 56·0 (46·0–67·0) | 15 (8%) CAD  58 (30%) HTN | 44 (23%) Heart failure  33 (17%) ACI | N/A | 72/172 (42%) | 54 (28.2%) Deceased |
|  |  |  |  | 54  Deceased | 38 (70%) | 69·0 (63·0–76·0) | 13 (24%) CAD  26 (48%) HTN | 28 (52%) Heart failure  32 (59%) ACI | N/A | 44 (81%) | Deceased |
|  |  |  |  | 137  Recovered | 81 (59%) | 52·0 (45·0–58·0) | 2 (1%) CAD  32 (23%) HTN | 16 (12%) Heart failure  1 (1%) ACI | N/A | 28/118 (24%) | Survived |
| 15 | Gao L. 2020 | Respiratory research | Retrospective observational | 54  Total | 44.4% | 60.4 ± 16.1 | 12 (22.2%) HTN  9 (16.7%) CAD | N/A | N/A | N/A | 18 (33.3%) Deceased |
|  |  |  |  | 24  NT-proBNP≤88.64 pg/ml | 33.3% | 51.6 ± 13.9 | 2 (8.3%) HTN  1 (4.2%) CAD |  |  |  | 0 Deceased |
|  |  |  |  | 30  NT-proBNP> 88.64 pg/ml | 53.3% | 67.4 ± 14.4 | 10 (33.3%) HTN  8 (26.7%) CAD |  |  |  | 18 (60%) Deceased |
| 16 | Guo T. 2020 | JAMA cardiology | Case series | 187  Total | 48.7% | 58.50 (14.66) | 8 (15.4) Cardiomyopathy  61 (32.6%) HTN  21(11.2%) CAD | 11 (5.9%) Arrhythmia (VT/VF) | 18 (14.6%) AKI | 0.43 (0.19-2.66) μg/mL | 43 (23%) Deceased |
|  |  |  |  | 135  Normal TnT level | 42.2% | 53.35 (13.22) | 0 Cardiomyopathy  28 (20.7%) HTN  4 (3.0%) CAD | 2 (1.5%) Arrhythmia (VT/VF) | 4 (4.7%)  AKI | 0.29 (0.17-0.60) μg/mL | 12 (8.9%) Deceased |
|  |  |  |  | 52  High TnT level | 65.4% | 71.40 (9.43) | 8 (15.4) Cardiomyopathy  33 (63.5%) HTN  17 (32.7%) CAD | 9 (17.3%) Arrhythmia (VT/VF) | 14 (36.8%) AKI | 3.85 (0.51-25.58) μg/mL | 31 (59.6%) Deceased |
| 17 | He XW. 2020 | Zhonghua xin xue guan bing za zhi | Retrospective cohort | 26 Deceased | 16 (61.5%) | 70.0 (62.5, 76.5) | 5 (19.2%) CVD  18 (64.3%) HTN | 18 (69.2%) Myocardial injury | N/A | N/A | Deceased |
|  |  |  |  | 28  Survival | 18 (64.3%) | 66.5 (56.0, 71.8) | 3 (10.7%) CVD  12 (42.9%) HTN | 6 (21.4%) Myocardial injury |  |  | Survived |
|  |  |  |  | 24  With myocardial injury | 17 (70.8%) | 69.5 (63.3, 74.8) | 1 (4.2%) CVD  10 (41.7%) HTN | All |  |  | 18 (75%)  Deceased |
|  |  |  |  | 30  without myocardial injury | 17(56.7%) | 67.0 (57.0, 74.3) | 7 (23.3%) CVD  14 (46.7%) HTN | None |  |  | 8 (26.7%) Deceased |
| 18 | Shi S. 2020 | JAMA cardiology | cohort study | 416  Total | 49.3% | 64 (21-95) | 127 (30.5%) HTN  44 (10.6%) CAD  17 (4.1%) Heart failure | 19.7% ACI  14 (3.4%) Chest pain | 8 (1.9%) AKI | N/A | 57 (13.7%) Deceased |
|  |  |  |  | 82  With cardiac injury | 53.7% | 74 (34-95) | 49 (59.8%) HTN  24 (29.3%) CAD  12 (14.6%) Heart failure | 11 (13.4%) Chest pain | 7 (8.5%) AKI |  | 42 (51.2%) Deceased |
|  |  |  |  | 334  Without cardiac injury | 48.2% | 60 (21-90) | 78 (23.4%) HTN  20 (6.0%) CAD  5 (1.5%) Heart failure | 3 (0.9%) Chest pain | 1 (0.3%) AKI |  | 15 (4.5%) Deceased |
| 19 | Du RH. 2020 | Annals of the American Thoracic Society | Observational study | 109  Total | 67.9% | 70.7±10.9 | 65 (59.6%) HTN  37 (33.9) CVD or  cerebrovascular diseases | 52 (47.7%) ACI | N/A | 85 (78.0%) | 109 (100%) Deceased |
|  |  |  |  | 51  ICU | 70.6% | 68.4±9.7 | 29 (56.9%) HTN  15 (29.4) CVD or  cerebrovascular diseases | 25 (49.0%) ACI |  | 44 (86.3%) |  |
|  |  |  |  | 58  Non-ICU | 65.5% | 72.7±11.6 | 36 (62.1%) HTN  22 (37.9) CVD or  cerebrovascular diseases | 27 (46.6%) ACI |  | 44 (86.3%) |  |
| 20 | Huang C. 2020 | Lancet | Cohort | 41 Total | 30 (73%) | 49.0 (41.0-58.0) | 6 (15%) CVD  6 (15%) HTN | 5 (12%) ACI  3 (7%) Shock | 3 (7%) | 3.4 (1.1-9.1) mg/L | 6 (15%) Deceased  28 (68%) Recovered  7 (17%) Remained hospitalized |
|  |  |  |  | 13  ICU | 11 (85%) | 49.0 (41.0-61.0) | 3 (23%) CVD  2 (15%) HTN | 4 (31%) ACI  3 (23%) Shock | 3 (23%) | 3.3 (3.0-163.0) mg/L | 5(38%) Deceased  7 (54%) Recovered  1 (8%) remained hospitalized |
|  |  |  |  | 28  Non-ICU | 19 (68%) | 49.0 (41.0-57.5 | 3 (11%) CVD  4 (14%) HTN | 1 (4%) ACI | 0 | 3.5 (0.7-5.4)  mg/L | 1 (6%) Deceased  21 (75%) Recovered  6 (21%) remained hospitalized |
| 21 | Lei S. 2020 | EClinicalMedicine | Retrospective analysis | 34  Total | 41.2% | 55 (43-63) | 13 (38.2%) HTN  7 (20.6%) CVD | 10 (29.4%) Shock  8 (23.5%) Arrhythmia  5 (14.7%) ACI | 2 (5.9%) | N/A | 7 (20.6%)  Deceased |
|  |  |  |  | 15  ICU | 33.3% | 55 (44-74) | 9 (60%) HTN  6 (40%) CVD | 8 (53.3%) Shock  5 (33.3) Arrhythmia  5 (33.3%) ACI | 2 (13.3%) |  |  |
|  |  |  |  | 19  Non-ICU | 47.4% | 47 (29-58) | 4 (21.1%) HTN  1 (5.3%) CVD | 2 (10.5%) Shock  3 (15.8%) Arrhythmia  0 ACI | 0 |  |  |
| 22 | Wang D. 2020 | JAMA - Journal of the American Medical Association | Retrospective case series | 138  Total | 54.3% | 56 (42-68) | 20 (14.5%) CAD  43 (31.2%) HTN | 23 (16.7&) Arrhythmia  10 (7.2%) ACI  12 (8.7%) Stroke | 5 (3.6%) AKI | 203 (121-403)  mg/L | 6 Deceased |
|  |  |  |  | 36  ICU | 61.1% | 66 (57-78) | 9 (25.0%) CAD  21 (58.3%) HTN | 16 (44.4%) Arrhythmia  8 (22.2%) ACI  11 (30.6%) Stroke | 3 (8.3%) AKI | 414 (191-1324)  mg/L | N/A |
|  |  |  |  | 102  Non-ICU | 52% | 51 (37-62) | 11 (10.8%) CAD  22 (21.6%) HTN | 7 (6.9%) Arrhythmia  2 (2.0%) ACI  1 (1.0%) Stroke | 2 (2.0%) AKI | 166 (101-285)  mg/L |  |
| 23 | Chen C. 2020 | Zhonghua xin xue guan bing za zhi | Retrospective cohort | 150  Total | 84 (56%) | 59 (16) | N/A | N/A | N/A | N/A | 11 (7.3%)  Deceased |
|  |  |  |  | 24  Critical | 18 (75%) | 68.5 (13.6) | 6 (25.0%) CVD  14 (58.3%) HTN | 15 (62.5%) ACI |  |  | 10 (41.7%) Deceased |
|  |  |  |  | 126  Non-critical | 66 (52.3%) | 57.1 (15.6) | 3(2.4%) CVD  35 (27.8%) HTN | 7 (5.6%) ACI |  |  | 1 (0.8%) Deceased |
| 24 | Guan W. 2020 | NEJM | Retrospective analysis | 1099  Total | 637/1096(58%) | 47.0 (35.0-58.0) | 165 (15.0%) HTN  27 (2.5%) CAD | N/A | N/A | 260/560 (46.4%) | N/A |
|  |  |  |  | 926  Non-severe | 537/923 (58%) | 45.0 (34.0-57.0) | 124 (13.4) HTN  17 (1.8%) CAD |  |  | 165/451 (43.2%) |  |
|  |  |  |  | 173  Severe | 100/173  (58%) | 52.0 (40.0-65.0) | 41 (23.7%) HTN  10 (5.8%) CAD |  |  | 65/109 (59.6%) |  |
|  |  |  |  | 67  Primary compost endpoint present | 45/67 (67%) | 63.0 (53.0-71.0) | 24 (35.8%) HTN  6 (9.0%) CAD |  |  | 34/49 (69.4%) |  |
|  |  |  |  | 1032  Primary compost endpoint  not present | 592/1029 (57%) | 46.0 (35.0-57.0) | 141 (13.7%) HTN  21 (2.0%) CAD |  |  | 226/511 (44.2%) |  |
| 25 | Han H. 2020 | Journal of medical virology | Retrospective cohort | 198  Mild | 71(35.8%) | 58.95 (10.80) | N/A | 10 (5.05%) ACI | N/A | N/A | 2 Deceased |
|  |  |  |  | 60  Severe | 21 (35%) | 58.97 (14.38) | N/A | 14 (23.33%) ACI |  |  | 19 Deceased |
|  |  |  |  | 15  Critical | 5(33.3%) | 57.27(17.25) | N/A | 3(20.00%) ACI |  |  | 3 Deceased |
| 26 | Li R. 2020 | Journal of Clinical Virology | Retrospective analysis | 225  Total | N/A | N/A | N/A | N/A | N/A | N/A | N/A |
|  |  |  |  | 188  Severe |  |  | 86 (45.95%) HTN |  |  |  |  |
|  |  |  |  | 37  Non- severe |  |  | 6 (15.96%) HTN |  |  |  |  |
| 27 | Li Xia. 2020 | Journal of Allergy and Clinical Immunology | Retrospective analysis | 548  Total | 50.9% | 60 (48-69) | 166 (30.3%) HTN  34 (6.2%) CAD | 41 (7.5%) Chest pain  162 (38.1%) Chest tightness  119 (21.7%) ACI | 95 (17.3%) | 227/501 (45.3%) | 90/545 (16.5%) Deceased  287/545 (52.7%) Discharged  168/545 (30.8%) Remained hospitalized |
|  |  |  |  | 279  Non-severe | 45.2% | 56 (44-66) | 62 (22.2%) HTN  6 (2.2%) CAD | 25 (9%) Chest pain  86 (42.2%) Chest tightness  25 (9%) ACI | 33 (11.8%) | 78/254 (31.1%) | 3/277 (1.1%) Deceased  202/277 (72.9%) Discharged  72/277 (26.0%) remained hospitalized |
|  |  |  |  | 269  Severe | 56.9% | 65 (54-72) | 104 (38.7%) HTN  28 (10.4%) CAD | 16 (6%) Chest pain  76 (38.1%) Chest tightness  94 (34.9%) ACI | 62 (23.0%) | 149/247 (56.4%) | 87/268 (32.5%) Deceased  85/268 (31.7%) Discharged  96/268 (35.8%) remained hospitalized |
| 28 | Peng Y.D. 2020 | Chinese Journal of Cardiovascular Diseases | Retrospective case series | 112  Total | 47.32% | 62.0 (55.0, 67.0) | 62 (55.36%) CAD  40 (35.71%) Heart failure  92 (82.14%) HTN | 38 (33.93%) Chest pain  3 (2.67%) Acute myocardial infarction🡪 cause of death  2 (1.78%) Heart failure🡪 cause of death | N/A | N/A | 17 (15.18%) Deceased |
|  |  |  |  | 16  Severe | 56.25% | 57.5 (54.0, 63.00) | 10 (62.50%) CAD  9 (56.25%) Heart failure  10 (62.50%) HTN | 4 (25.00%) Chest pain |  |  | 11 (64.71%) Deceased |
|  |  |  |  | 96  Non-severe | 45.83% | 62.0 (55.0, 67.5) | 52 (54.17%) CAD  31 (32.29%) Heart failure  82 (85.42%) HTN | 34 (35.53%) Chest pain |  |  | 6 (35.29%) Deceased |
|  |  |  |  | 95  Cured | N/A | N/A | 47 (49.47%) CAD  27 (28.42%) Heart failure  77 (81.05%) HTN | N/A |  |  | Survived |
|  |  |  |  | 17  Deceased | N/A | N/A | 15 (88.24%) CAD  13 (76.47%) Heart failure  15 (88.24%) HTN |  |  |  | 100% Deceased |
| 29 | Wan S. 2020 | Journal of Medical Virology | Retrospective case series | 135  Total | 53.3% | 47(36-55) | 7 (5.2%) CAD  13 (9.6%) HTN | 10(7.4%) ACI  1(0.7%) Shock | 5(3.7%) AKI | 0.4(0.2-0.6)  mg/L | 1(0.7%) Deceased |
|  |  |  |  | 95  Mild | 54.7% | 44(33-49) | 1(1%) CAD  9(9.4%) HTN | 8(8.4%) ACI  0 Shock | 4(4%) AKI | 0.3(0.2-0.5)  mg/L | Survived |
|  |  |  |  | 40  Severe | 52.5% | 56(52-73) | 6(15%) CAD  4(10%) HTN | 2(5%) ACI  1(2.5%) shock | 1(2.5%) AKI | 0.6(0.4-1.1)  mg/L | 1(2.5%) Deceased |
| 30 | Zhang J.-J. 2020 | Allergy: European Journal of Allergy and Clinical Immunology | Retrospective cohort | 140  Total | 50.7% | 57 (25-87) | 5 (3.6%) Arrhythmia  7 (5.0%) CAD  42 (30%) HTN  3 (2.1%) Stroke | N/A | N/A | 35/81 (43.2%) | N/A |
|  |  |  |  | 82  Non-severe | 46.3% | 51.5 (26-78) | 1 (1.2%) Arrhythmia  3 (3.7%) CAD  20 (24.4%) HTN  1 (1.2%) Stroke |  |  | 12/43 (27.9%) |  |
|  |  |  |  | 58  Severe | 56.9% | 64 (25-87) | 4 (6.9%) Arrhythmia  4 (6.9%) CAD  22 (37.9%) HTN  2 (3.4%) Stroke |  |  | 23/38 (60.5%) |  |
| 31 | Zheng F. 2020 | Eur Rev Med Pharmacol Sci | Retrospective analysis | 161  Total | 49.7% | 45 (33.5, 57) | 22 (13.7%) HTN  4 (2.5%) CAD | N/A | N/A | N/A | N/A |
|  |  |  |  | 131  Non-severe | 50.4% | 40 (31, 51) | 10 (7.6%) HTN  2 (1.5%) CAD |  |  |  |  |
|  |  |  |  | 30  Severe | 46.7% | 57 (46.5, 66) | 12 (40%) HTN  2 (6.7%) CAD |  |  |  |  |
| 32 | Xu XW. 2020 | BMJ | Retrospective case series | 62  Total | 56% | 41 (32-52) | 5 (8%) HTN | N/A | 1 (2%) Renal diseases | 0.2 (0.2-0.5) mg/L | Survived |
|  |  |  |  | 33  Time since symptom onset>10 days | 58% | 45 (37-54) | 4 (12%) HTN |  | 0 Renal diseases | 0.2 (0.2-0.6) mg/L | Survived |
|  |  |  |  | 29  Time since symptom onset<10 days | 55% | 39 (31-50) | 1 (3%) HTN |  | 1 (3%) Renal diseases | 0.2 (0.2-0.4) mg/L | Survived |
| 33 | Lian J. 2020 | Clinical Infectious Diseases | Retrospective study | 652  Age<60 | 53.53% | 41.15±11.38 | 5 (0.77%) Heart failure  73 (11.20%) HTN | N/A | 10 (1.53%) AKI | N/A | 291 Discharged/ Stay in hospital |
|  |  |  |  | 136  Age≥60 | 42.65% | 68.28±7.314 | 6 (4.41 %) Heart failure  53 (38.97%) HTN |  | 3 (2.21%) AKI |  | 31 Discharged/ Stay in hospital |
| 34 | Young, Barnaby Edward 2020 | JAMA | Descriptive case series | 18  Total | 50% | 47 (31-73) | 5 (27.7%) HTN | N/A | N/A | N/A | Survived |
|  |  |  |  | 12  Did not require  supplemental O2 | 58% | 37 (31-56) | 1 (8%) HTN |  |  |  |  |
|  |  |  |  | 6  Required  supplemental O2 | 33% | 56 (47-73) | 4 (67%) HTN |  |  |  |  |
| 35 | Zhang P. 2020 | Circulation research | Retrospective study | 3430  Total | 48.8% | 57(45-65) | 178 (5.2%) CAD | N/A | N/A | 1055/2567 (41.1%) | N/A |
|  |  |  |  | 1128  HTN | 53.5% | 64(56-69) | 131 (11.6) CAD |  |  | 459/892 (51.4%) |  |
|  |  |  |  | 188  ACEI/ARB | 53.2% | 64 (55-68) | 29 (15.4%) CAD |  |  | 67/149 (45.0%) |  |
|  |  |  |  | 557  Non-ACEI/ARB | 49.7% | 64 (57-68) | 56(10.1%) CAD |  |  | 240/445 (53.9%) |  |
|  |  |  |  | 2302  Non-HTN | 46.6% | 52(40-62) | 47(2.0) CAD |  |  | 596/1675 (35.6%) |  |
| Abbreviations:  HTN: Hypertension, CVD: Cardiovascular disease, CAD: Coronary artery disease, CHF: Congestive heart failure, ACI: Acute cardiac injury, AKI: Acute kidney injury, ACS: Acute coronary syndrome | | | | | | | | | | | |
