# Supplementary material for: Cardiovascular disease in COVID-19: a systematic review and meta-analysis of 10,898 patients and proposal of a triage risk stratification tool
Source: Egypt Heart J. 2020 Jul 13;72:41. doi: 10.1186/s43044-020-00075-z (PMC7356124; doi:10.1186/s43044-020-00075-z)
Supplement: Supplementary file 3 — Additional file 3: Supplementary Material 3 (S3) Supplementary Table2. Inflammatory and cardiac biomarkers in COVID-19 [file 43044_2020_75_MOESM3_ESM.docx]

**Supplementary Material 3 (S3)**

**Supplementary Table2. Inflammatory and cardiac biomarkers in COVID-19**

| No. | Author | No. of patients | Inflammatory markers  Median (interquartile range) and N. of patients (percentage) | Cardiac biomarkers  Median (interquartile range) and N. of patients (percentage)* |
| --- | --- | --- | --- | --- |
| 1 | Arentz M. 2020 | 21  Total | N/A | CK: 95 U/L (45-1290)  Tn: 3 (14.0%) |
| 2 | Chen N. 2020 | 99  Total | IL-6: 51 (52%)  ESR: 49.9 mm/h (23.4) and 84 (85%)  Serum Ferritin: 808.7 ng/mL (490.7) and 62 (63%)  CRP: 51·4 mg/L (41·8) and 63/73 (86%) | CK: 85·0 U/L (51·0–184·0) and 13 (13%) and 23 (23%) 🠋  LDH: 336·0 U/L (260·0–447·0) and 75 (76%)  Myoglobin: 15 (15%) |
| 3 | Du Y. 2020 | 85  Total | CRP: 107.259 ± 117.215 mg/L and 78 (91.8%) | CK: 298.0 ± 401.8 U/L and 31 (36.5%)  LDH: 645.8 ± 596.9 U/L and 70 (82.4%) |
| 6 | Li X. 2020 | 25  Total | CRP: 91.1 mg/L (55.55 – 146.3) | hs-cTnI: 316 ng/mL (57 – 5420) and 11 (73.3%)  NT-proBNP: 2450 pg/mL (881 - 7992) and 16 (94.1%) |
| 8 | Sun C. 2020 | 150  Total | ESR: 32 ± 22 mm/h and 86 (57%)  CRP: 23 ± 8 mg/L and 66 (44%) | CK: 88 ± 67 U/L and 11 (7%)  LDH: 245 ± 125 U/L and 47 (31%) |
| 9 | Chen T. 2020 | 274  Total | IL-6: 22.0 pg/mL (6.1-51.8) and 119/163 (73%)  TNF-α: 8.6 pg/mL (7.0-12.2) and 93/163 (57%)  ESR: 32.5 mm/h (17.3-53.8)  Serum Ferritin: 669.7 μg/L (388.8-1494.6)  CRP: 53.4 mg/L (18.6-113.0) and 80/243 (33%) | hs-cTnI: 8.7 pg/mL (2.9-33.6) and 83/203 (41%)  NT-proBNP: 267.0 pg/mL (48.0-821.0) and 85/173(49%)  CK: 109.0 U/L (53.5-188.0) |
|  |  | 113  Deceased | IL-6: 72.0 pg/mL (35.6-146.8) and 53/53 (100%)  TNF-α: 11.8 pg/mL (8.6-17.6) and 41/53 (77%)  ESR: 38.5 mm/h (20.5-62.8)  Serum Ferritin: 1418.3 μg/L (915.4-2236.2)  CRP: 113.0 mg/L (69.1-168.4) and 59/98 (60%) | hs-cTnI: 40.8 pg/mL (14.7-157.8) and 68/94(72%)  NT-proBNP: 800.0 pg/mL (389.8-1817.5) and 68/80 (85%)  CK: 189.0 U/L (94.5-374.5) |
|  |  | 161 Recovered | IL-6: 13.0 pg/mL (4.0-26.2) and 66/110 (60%)  TNF-α: 7.9 pg/mL (6.7-9.6) and 52/110 (47%)  ESR: 28.0 mm/h (15.8-45.0)  Serum Ferritin: 481.2 μg/L (265.1-871.5)  CRP: 26.2 mg/L (8.7-55.8) and 21/145 (14%) | hs-cTnI: 3.3 pg/mL (1.9-7.0) and 15/109(14%)  NT-proBNP: 72.0 pg/mL (20.0-185.0) and 17/93 (18%)  CK: 72.0 U/L (20.0-185.0) |
| 10 | Du RH. 2020 | 179  Total | CRP: 39.8 mg/L (20.6‒97.8) | N/A |
|  |  | 21  Deceased | CRP: 86.4 mg/L (37.9‒105.5) | cTnI: 13 (61.5%)  Myoglobin: 14 (64.3%)  BNP: 20 (94.1%) |
|  |  | 158  Recovered | CRP: 36.0 mg/L (19.3‒91.0) | cTnI: 27 (17.9%)  Myoglobin: 29 (18.4%)  BNP: 107 (67.6%) |
| 11 | Ruan Q. 2020🕇 | 68  Deceased | IL-6: 11.4 pg/mL (8.5)  CRP: 126.6 mg/L (106.3)  Serum Ferritin: 1297.6 ng/mL (1030.9) | CK: 319.4 U/L (838.5)  LDH: 905.8 U/L (2619.1)  cTn: 30.3 pg/mL (151.0)  Myoglobin: 258.9 ng/mL (307.6) |
|  |  | 82  Recovered | IL-6: 6.8 pg/ml (3.61)  CRP: 34.1 mg/L (54.5)  Serum Ferritin: 614.0 ng/mL (752.2) | CK: 231.7 U/L (862.3)  LDH: 297.9 U/L (110.4)  cTn: 3.5 pg/mL (6.2)  Myoglobin: 77.7 ng/mL (136.1) |
| 12 | Zhou F. 2020 | 191  Total | IL-6: 7·4 pg/mL (5·3–10·8)  Serum Ferritin: 722.0 μg/L (377.2–1435.3) and 102/128 (80%) | hs-cTnI: 4·1 ng/mL (2·0–14·1) and 24/145 (17%)  LDH: 300·0 U/L (234·0–407·0) and 123/184 (67%)  CK: 21·5 U/L (13·0–72·4) and 22/168 (13%) |
|  |  | 54  Deceased | IL-6: 11·0 pg/mL (7·5–14·4)  Serum Ferritin: 1435.3 μg/L (728.9–2000.0) and 44/46 (96%) | hs-cTnI: 22·2 ng/mL (5·6–83·1) and 23/50 (46%)  LDH: 521·0 U/L (363·0–669·0) and 53 (98%)  CK: 39·0 U/L (19·5–151·0) and 11/52 (21%) |
|  |  | 137  Recovered | IL-6: 6·3 pg/mL (5·0–7·9)  Serum Ferritin: 503.2 μg/L (264.0–921.5) and 58/82 (71%) | hs-cTnI: 3·0 ng/mL (1·1–5·5) and 1/95 (1%)  LDH: 253·5 U/L (219·0–318·0) and 70/130(54%)  CK: 18·0 U/L (12·5–52·1) and 11/116 (9%) |
| 13 | Gao L. 2020 | 54  Total | CRP: 34.8 mg/L (5.3–61.0) | NT-proBNP: 137.30 pg/ml (39.64–494.98)  Myoglobin: 39.28 ng/mL (26.26–86.84)  CK-MB: 1.04 ug/L (0.65–2.27)  hs-cTnI: < 0.006 ng/mL (< 0.006–0.022) |
|  |  | 24  NT-proBNP≤88.64 pg/ml | CRP: 7.6 mg/L (5.0–34.8) | NT-proBNP: 37.28 pg/ml (22.28–61.74)  Myoglobin: 25.35 ng/mL (14.04–35.20)  CK-MB: 0.63 ug/L (0.37–0.79)  hs-cTnI: < 0.006 ng/mL (< 0.006- < 0.006) |
|  |  | 30  NT-proBNP> 88.64 pg/ml | CRP: 54.3 mg/L (14.3–117.9) | NT-proBNP: 420.40 pg/ml (199.63–919.88)  Myoglobin: 82.53 ng/mL (34.55–123.96)  CK-MB: 1.90 ug/L (1.08–3.78)  hs-cTnI: 0.021 ng/mL (< 0.006–0.136) |
| 14 | Guo T. 2020 | 187  Total | CRP: 4.04 mg/dL (1.64-8.14) | NT-proBNP: 268.4 pg/mL (75.3-689.1)  CK-MB: 1.14 ng/ml (0.66-2.95)  Myoglobin: 38.5 μg/L (21.0-78.0) |
|  |  | 135  Normal TnT level | CRP: 3.13 mg/dL (1.24-5.75) | NT-proBNP: 141.4 pg/mL (39.3-303.6)  CK-MB: 0.81 ng/ml (0.54-1.38)  Myoglobin: 27.2 μg/L (21.0-49.8) |
|  |  | 52  High TnT level | CRP: 8.55 mg/dL (4.87-15.165) | NT-proBNP: 817.4 pg/mL (336.0-1944.0)  CK-MB: 3.34 ng/ml (2.11-5.80)  Myoglobin: 128.7 μg/L (65.8-206.9) |
| 15 | He XW. 2020 | 26 Deceased | ESR: 43.0 mm/h (17.8, 77.8) | NT-proBNP: 852 ng/mL (400.0, 2315.3) |
|  |  | 28  Survival | ESR: 33.0 mm/h (23.5, 60.5) | NT-proBNP: 197.0 ng/mL (115.3, 631.0) |
|  |  | 24  With myocardial injury | ESR: 31.0 mm/h (17.5, 62.0)  CRP: 135.0 mg/L (78.8, 195.1) | NT-proBNP: 971.0 ng/mL (504.0, 2106.0) |
|  |  | 30  without myocardial injury | ESR: 40.0 mm/h (25.0, 65.0)  CRP: 62.9 mg/L (28.8, 109.3) | NT-proBNP: 201.0 ng/mL (127.3, 670.3) |
| 16 | Shi S. 2020 | 416  Total | CRP: 4.5 mg/dL (1.4-8.5) | TnI: <0.006 μg/L (<0.006-0.02) and 50 (12%)  NT-proBNP: 219 pg/mL (73-699)  CK-MB: 1.0 ng/mL (0.7-2.0)  Myoglobin: 47 μg/L (28-93) |
|  |  | 82  With cardiac injury | CRP: 10.2 mg/dL (6.4-17.0) | TnI: 0.19 μg/L (0.08-1.12)  NT-proBNP: 1689 pg/mL (698-3327)  CK-MB: 3.2 ng/mL (1.8-6.2)  Myoglobin: 128 μg/L (68-305) |
|  |  | 334  Without cardiac injury | CRP: 3.7 mg/dL (1.0-7.3) | TnI: <0.006 μg/L (<0.006-0.009)  NT-proBNP: 139 pg/mL (51-335)  CK-MB: 0.9 ng/mL (0.6-1.3)  Myoglobin: 39 μg/L (27-65) |
| 17 | Du RH. 2020 | 109  Total | CRP: 85.7±57.3 mg/L and 104 (95.4%) | Cardiac TnI: 0.0 ng/mL (0.0‒0.1) and 52 (47.7%)  CK-MB: 39 (35.8%)  Myoglobin: 68.3 μg/L (36.6–141.1) and 42 (38.5%)  NT-proBNP: 582.0 ng/L (307.0–1097.5) and 34/45 (75.6%) |
|  |  | 51  ICU | CRP: 87.1±63.0 mg/L and 48 (94.1%) | Cardiac TnI: 0.1 ng/mL (0.0‒0.8) and 25 (49.0%)  CK-MB: 15 (29.4%)  Myoglobin: 71.4 μg/L (37.3–153.9) and 23 (45.1%)  NT-proBNP: 480.0 ng/L (164.0–1046.5) and 18/28 (64.3%) |
|  |  | 58  Non-ICU | CRP: 84.5±52.3 mg/L and 56 (96.6%) | Cardiac TnI: 0.0 ng/mL (0.0‒0.0) and 27 (46.6%)  CK-MB: 24 (41.4)  Myoglobin: 68.1 μg/L (35.5–133.7) and 19 (32.8%)  NT-proBNP: 722.0 ng/L (498.5-1335.0) and 16/17 (94.1%) |
| 18 | Huang C. 2020 | 41 Total | N/A | hs-cTnI: 3·4 pg/mL (1·1–9·1) and 5/41 (12%)  CK: 132·5 U/L (62·0–219·0) and 27/40 (68%)  LDH: 286·0 U/L (242·0–408·0) and 11/40 (28%) |
|  |  | 13  ICU |  | hs-cTnI: 3·3 pg/mL (3·0–163·0) and 4/13 (31%)  CK: 132·0 U/L (82·0–493·0) and7/13 (54%)  LDH: 400·0 U/L (323·0–578·0) and 1/13 (8%) |
|  |  | 28  Non-ICU |  | hs-cTnI: 3·5 pg/mL (0·7–5·4) and 1/28 (4%)  CK: 133·0 U/L (61·0–189·0) and 20/27 (74%)  LDH: 281·0 U/L (233·0–357·0) and 10/27 (37%) |
| 19 | Lei S. 2020 | 34  Total | CRP: 30.3 mg/L (8.4-74.3) | LDH: 209 U/L (191-230)  CK: 61 U/L (43-94) |
|  |  | 15  ICU | CRP: 29.6 mg/L (12.5-86.6) | LDH: 218 U/L (188-230)  CK: 70 U/L (47-163) |
|  |  | 19  Non-ICU | CRP: 24.8 mg/L (7.7-72.3) | LDH: 207 U/L (192-231)  CK: 61 U/L (31-89) |
| 20 | Wang D. 2020 | 138  Total | N/A | TnI: 6.4 pg/mL (2.8-18.5)  LDH: 261 U/L (182-403)  CK: 92 U/L (56-130)  CK-MB: 14 U/L (10-18) |
|  |  | 36  ICU |  | TnI: 11.0 pg/mL (5.6-26.4)  LDH: 435 U/L (302-596)  CK: 102 U/L (62-252)  CK-MB: 18 U/L (12-35) |
|  |  | 102  Non-ICU |  | TnI: 5.1 pg/mL (2.1-9.8)  LDH: 212 U/L (171-291)  CK: 87 U/L (54-121)  CK-MB: 13 U/L (10-14) |
| 21 | Chen C. 2020 | 150  Total | N/A | N/A |
|  |  | 24  Critical | CRP: 84.9 mg/L (56.8, 148.2) | cTnI: 4.5 ng/L (2.7, 10.0) and 15 (62.5%)  NT-proBNP: 83 ng/L (28,232) and 19 (79.2%) |
|  |  | 126  Non-critical | CRP: 30.9 mg/L (7.1, 66.9) | cTnI: 68.5 ng/L (9.3, 693.3) and 7 (5.6%)  NT-proBNP: 1030 ng/L (339, 2276) and 28 (22.2%) |
| 22 | Guan W. 2020 | 1099  Total | CRP: 481/793 (60.7%) | LDH: 277/675 (41.0%)  CK: 90/657 (13.7%) |
|  |  | 926  Non-severe | CRP: 371/658 (56.4%) | LDH: 205/551 (37.2%)  CK: 67/536 (12.5%) |
|  |  | 173  Severe | CRP: 110/135 (81.5%) | LDH: 72/124 (58.1%)  CK: 23/121 (19.0%) |
|  |  | 67  Primary compost endpoint present | CRP: 41/45 (91.1%) | LDH: 31/44 (70.5%)  CK: 12/46 (26.1%) |
|  |  | 1032  Primary compost endpoint  not present | CRP: 440/748 (58.8%) | LDH: 246/631 (36.0%)  CK: 78/611 (12.8%) |
| 23 | Han H. 2020 | 198  Mild | N/A | CK-MB: 0.91 ng/mL (0.61~1.41) and 192 (96.97%)  ultra-TnI: 0.01 ng/mL (0.01~0.01) and 10 (5.05%)  NT-proBNP: 113.65 pg/mL (45.92~274.23) and 14 (7.07%)  Myoglobin: 34.66 μg/L (26.46~54.54) and 11 (5.56%) |
|  |  | 60  Severe |  | CK-MB: 1.10 ng/mL (0.76~2.12) and 57 (95.00%)  ultra-TnI: 0.01 ng/mL (0.01~0.04) and 14 (23.33%)  NT-proBNP: 290.85 pg/mL (106.13~958.98) and 15 (25.00%)  Myoglobin: 57.73 μg/L (37.43~100.71) and 14 (23.33%) |
|  |  | 15  Critical |  | CK-MB: 0.97 ng/mL (0.32~2.37) and 14 (93.33%)  ultra-TnI: 0.01 ng/mL (0.01~0.03) and 3(20.00%)  NT-proBNP: 224.50 pg/mL (91.73~3615) and 5(33.33%)  Myoglobin: 75.34 μg/L (23.24~112.47) and 4 (26.67%) |
| 24 | Li Xia. 2020 | 548  Total | IL-1β: 51/306 (16.7%)  IL-2R: 164/309 (53.1%)  IL-6: 221/312 (70.8%)  IL-8: 24/309 (7.8%)  IL-10: 83/307 (27.0%)  TNF-α: 182/309 (58.9%)  ESR: 377/518 (72.8%)  CRP: 460/540 (85.2%)  Serum Ferritin: 211/313 (67.4%) | NT-proBNP: 92/335 (27.5%)  LDH: 133/534 (24.9%) |
|  |  | 279  Non-severe | IL-1β: 34/170 (20.0%)  IL-2R: 73/171 (42.7%)  IL-6: 107/175 (61.1%)  IL-8: 10/171 (5.9%)  IL-10: 34/170 (20.0%)  TNF-α: 89/171 (52.1%)  ESR: 179/264 (67.8%)  CRP: 205/272 (75.4%)  Serum Ferritin: 95/171 (55.9%) | NT-proBNP: 17/136 (13.3%)  LDH: 25/272 (9.2%) |
|  |  | 269  Severe | IL-1β: 17/136 (12.5%)  IL-2R: 91/138 (65.9%)  IL-6: 114/137 (83.2%)  IL-8: 14/137 (10.1%)  IL-10: 49/170 (35.8%)  TNF-α: 93/138 (67.4%)  ESR: 198/254 (78.0%)  CRP: 255/268 (95.2%)  Serum Ferritin: 116/142 (81.7%) | NT-proBNP: 75/199 (37.9%)  LDH: 108/262 (41.2%) |
| 25 | Peng Y.D. 2020 | 112  Total | N/A | N/A |
|  |  | 16  Severe | CRP: 106.98 mg/L (81.57, 135.76) | BNP: 20.35 ng/L (10.00, 77.05)  TnI: 9.40 ng/L (2.30, 10.30)  CK-MB: 13.00 U/L (8.00, 17.00)  LDH: 351.00 U/L (239.00, 413.50)  CK: 89.50 U/L (43.50, 234.00) |
|  |  | 96  Non-severe | CRP: 34.34 mg/L (9.55, 76.54) | BNP: 33.40 ng/L (21.90, 75.80)  TnI: 9.40 ng/L (4.75, 25.35)  CK-MB: 11.00 U/L (9.00, 15.00)  LDH: 290.00 U/L (227.50, 372.50)  CK: 77.00 U/L (42.00, 130.50) |
|  |  | 95  Cured | N/A | N/A |
|  |  | 17  Deceased |  | N/A |
| 26 | Wan S. 2020 | 135  Total | CRP: 10.5 mg/L (2.7-51.2) | LDH: 320.5 U/L (248.5-385.3) and 58 (43%)  CK: 82.2 U/L (56.3-146.3) and 10 (7.4%) |
|  |  | 95  Mild | CRP: 7.7 mg/L (1.9-31.1) | LDH: 212 U/L (179.5-259) and 28 (29%)  CK: 57 U/L (36.5-86.5) and 3 (3%) |
|  |  | 40  Severe | CRP: 91 mg/L (52.7-136.3) | LDH: 309 U/L (253.8-408.3) and 30 (75%)  CK: 82 U/L (56.3-146.2) and 7 (17.5%) |
| 27 | Zhang J.-J. 2020 | 140  Total | CRP: 34.2 mg/L (12.5-67.4) and 125/136 (91.9%) | CK: 72.5 U/L (52.2-115) and 4/60 (6.7%) |
|  |  | 82  Non-severe | CRP: 28.7 mg/L (9.5-52.1) and 72/81 (88.9%) | CK: 83.0 U/L (56.0-112.0) and 1/35 (2.8) |
|  |  | 58  Severe | CRP: 47.6 mg/L (20.6-87.1) and 47.6 (20.6-87.1%) | 66.0 U/L (38.5-144.0) and 3/25 (12.0) |
| 28 | Zheng F. 2020 | 161  Total | CRP: 17.9 mg/L (8.1, 36.7) and 121 (75.2%) | CK: 72.9 U/L (45.4, 72.2) and 17 (10.6%)  LDH: 177.1 U/L (141.0, 221.9) and 38 (23.6%) |
|  |  | 131  Non-severe | CRP: 15.4 mg/L (5.8, 24.9) and 91 (69.5%) | CK: 68.7 U/L (43.2, 111.3) and 8 (6.1%)  LDH: 162.0 U/L (133.7, 208.5) and 23 (17.6%) |
|  |  | 30  Severe | CRP: 52.2 (28.8, 75.1) and 30 (100%) | CK: 100.3 U/L (61.3, 398.6) and 9 (30%)  LDH: 226.2 U/L (193.5, 315.1) and 15 (50%) |
| 29 | Xu XW. 2020 | 62  Total | N/A | LDH: 205.0 U/L (184.0-260.5) and 17 (27%)  CK: 69.0 U/L (40.5-101.0) and 5 (8%) |
|  |  | 33  Time since symptom onset>10 days |  | LDH: 233.5 U/L (198.0-312.3) and 14 (42%)  CK: 60.0 U/L (40.0-106.8) and 2 (6%) |
|  |  | 29  Time since symptom onset<10 days |  | LDH: 194.5 U/L (166.3-213.8) and 3 (10)  CK: 71.4 U/L (60.3-90.5) and 3 (10%) |
| 30 | Lian J. 2020 | 652  Age<60 | CRP: 6.75 mg/L (2.0-16.9) | LDH: 204.0 U/L (165.0-255.0)  CK: 67.0 U/L (46.0-104.0) |
|  |  | 136  Age≥60 | CRP: 19.0 mg/L (5.6-44.7) | LDH: 244.0 U/L (206.0-311.0)  CK: 74.5 U/L (52.3-123.0) |
| 31 | Young, Barnaby Edward 2020 | 18  Total | CRP: 16.3 mg/L (0.9-97.5) | LDH: 512 U/L (285-796) |
|  |  | 12  Did not require  supplemental O2 | CRP: 11.1 mg/L (0.9-19.1) | LDH: 424 U/L (285-748) |
|  |  | 6  Required  supplemental O2 | CRP: 65.6 mg/L (47.5-97.5) | LDH: 550 U/L (512-796) |
| *Number of patients with elevated cardiac biomarker  🠋Number of patients with decreased levels of cardiac biomarker  🕇 In this study, values were reported as mean (SD) or median (IQR).  Abbreviations: CK: Creatine kinase, CK-MB: Creatine kinase myocardial band, BNP: Brain natriuretic peptide, NT-proBNP: N-terminal-pro hormone BNP, Tn: Troponin, TnI: Troponin I, cTn: Cardiac troponin, cTnI: Cardiac troponin I, hs-cTnI: High-sensitivity cardiac troponin I, LDH: Lactate dehydrogenase, TNF-α: Tumor necrosis factor alpha, IL: Interleukin, CRP: C-reactive protein, ESR: Erythrocyte sedimentation rate | | | | |
