# Supplementary material for: Cardiovascular disease in COVID-19: a systematic review and meta-analysis of 10,898 patients and proposal of a triage risk stratification tool
Source: Egypt Heart J. 2020 Jul 13;72:41. doi: 10.1186/s43044-020-00075-z (PMC7356124; doi:10.1186/s43044-020-00075-z)
Supplement: Supplementary file 4 — Additional file 4: Supplementary Material 4 (S4) Risk of bias assessment [file 43044_2020_75_MOESM4_ESM.docx]

**Supplementary Material 4 (S4)**

**S4. Risk of bias assessment**

| **Score** | **Outcome** | | | **Comparability** | | **Selection** | | | | **Study Author/ Year** |
| --- | --- | --- | --- | --- | --- | --- | --- | --- | --- | --- |
|  | **Non-response**  **rate** | **Same ascertainment** | **Ascertainment of**  **exposure** |  |  | **Definition of controls** | **Selection of controls** | **Representative of cases** | **Case definition adequacy** |  |
| 7 | * | * | * | * |  |  | * | * | * | Arentz M. 2020 |
| 8 | * |  | * | * | * | * | * | * | * | Chen C. 2020 |
| 7 | * | * | * | * |  | * | * |  | * | Chen N. 2020 |
| 8 |  | * | * | * | * | * | * | * | * | Chen T. 2020 |
| 7 |  | * | * | * | * |  | * | * | * | Deng Y. 2020 |
| 7 |  | * | * | * | * |  | * | * | * | Du RH. (survival)2020 |
| 8 | * | * | * | * |  | * | * | * | * | Du RH. 2020 |
| 8 | * | * | * | * | * | * | * |  | * | Du Y. 2020 |
| 8 | * | * | * | * | * | * | * |  | * | Gao L. 2020 |
| 6 | * |  | * | * | * |  | * |  | * | Garg S. 2020 |
| 8 | * | * | * | * | * |  | * | * | * | Grasselli G. 2020 |
| 8 | * | * | * | * | * | * | * |  | * | Guan W. 2020 |
| 7 | * | * | * | * | * |  | * |  | * | Guo T. 2020 |
| 5 | * |  | * | * |  |  | * |  | * | Han H. 2020 |
| 8 | * | * | * | * |  | * | * | * | * | He XW. 2020 |
| 5 |  | * | * | * | * |  | * |  |  | Huang C. 2020 |
| 8 | * | * | * | * | * |  | * | * | * | Lei S. 2020 |
| 7 | * |  | * | * |  | * | * | * | * | Li R. 2020 |
| 7 | * | * | * | * | * |  | * |  | * | Li X. 2020 |
| 8 | * | * | * | * | * |  | * | * | * | Li Xia. 2020 |
| 7 | * | * | * | * | * |  | * |  | * | Lian J. 2020 |
| 8 | * | * | * | * | * | * | * |  | * | Liu K. 2020 |
| 8 | * |  | * | * | * | * | * | * | * | Peng Y. D. 2020 |
| 8 | * | * | * | * | * |  | * | * | * | Ruan Q. 2020 |
| 7 | * | * | * | * | * |  | * |  | * | Shi S. 2020 |
| 5 |  | * | * | * |  |  | * |  | * | Sun C. 2020 |
| 7 | * |  | * | * | * |  | * | * | * | Wan S.2020 |
| 8 | * | * | * | * | * |  | * | * | * | Wang D. 2020 |
| 7 | * | * | * | * |  | * | * |  | * | Xu XW. 2020 |
| 7 | * |  | * | * | * | * | * |  | * | Xu X. 2020 |
| 7 | * | * | * | * | * |  | * |  | * | Young 2020 |
| 7 |  |  | * | * | * | * | * | * | * | Zhang J. 2020 |
| 6 | * |  | * | * |  |  | * | * | * | Zhang P. 2020 |
| 6 | * |  | * | * | * |  | * |  | * | Zheng F. 2020 |
| 8 | * | * | * | * | * |  | * | * | * | Zhou F. 2020 |
