# Supplementary material for: Cardiovascular disease in COVID-19: a systematic review and meta-analysis of 10,898 patients and proposal of a triage risk stratification tool
Source: Egypt Heart J. 2020 Jul 13;72:41. doi: 10.1186/s43044-020-00075-z (PMC7356124; doi:10.1186/s43044-020-00075-z)

Supplementary Material 5 (S5)

Forest plot of the pooled frequency analyses

Figure	Title	Page
**1**	Newly developed acute cardiac injury	**2**
**2**	Newly developed arrhythmia	**2**
**3**	Newly developed heart failure	**3**
**4**	Chest pain or chest tightness as initial presentation	**3**
**5**	Palpitation as initial presentation	**3**
**6**	Hypertension	**4**
**7**	Pre-existing cardiovascular diseases	**5**
**8**	Pre-existing heart failure	**5**
**9**	Diabetes mellitus	**6**
**10**	Elevated levels of NT-pro BNP	**7**
**11**	Elevated levels of cardiac troponins	**7**
**12**	Elevated levels of creatine kinase-MB	**8**
**13**	Elevated levels of creatine kinase	**8**
**14**	Elevated levels of D-dimer	**9**
**15**	Elevated levels of lactate dehydrogenase	**9**
**16**	Elevated levels of interleukin- 6	**10**
**17**	Elevated levels of C-Reactive Protein (CRP)	**10**
**18**	Elevated levels of erythrocyte sedimentation rate (ESR)	**11**
**19**	Elevated levels of ferritin	**11**

Figure 1. Forest plot of the pooled frequency analysis of newly developed acute cardiac injury

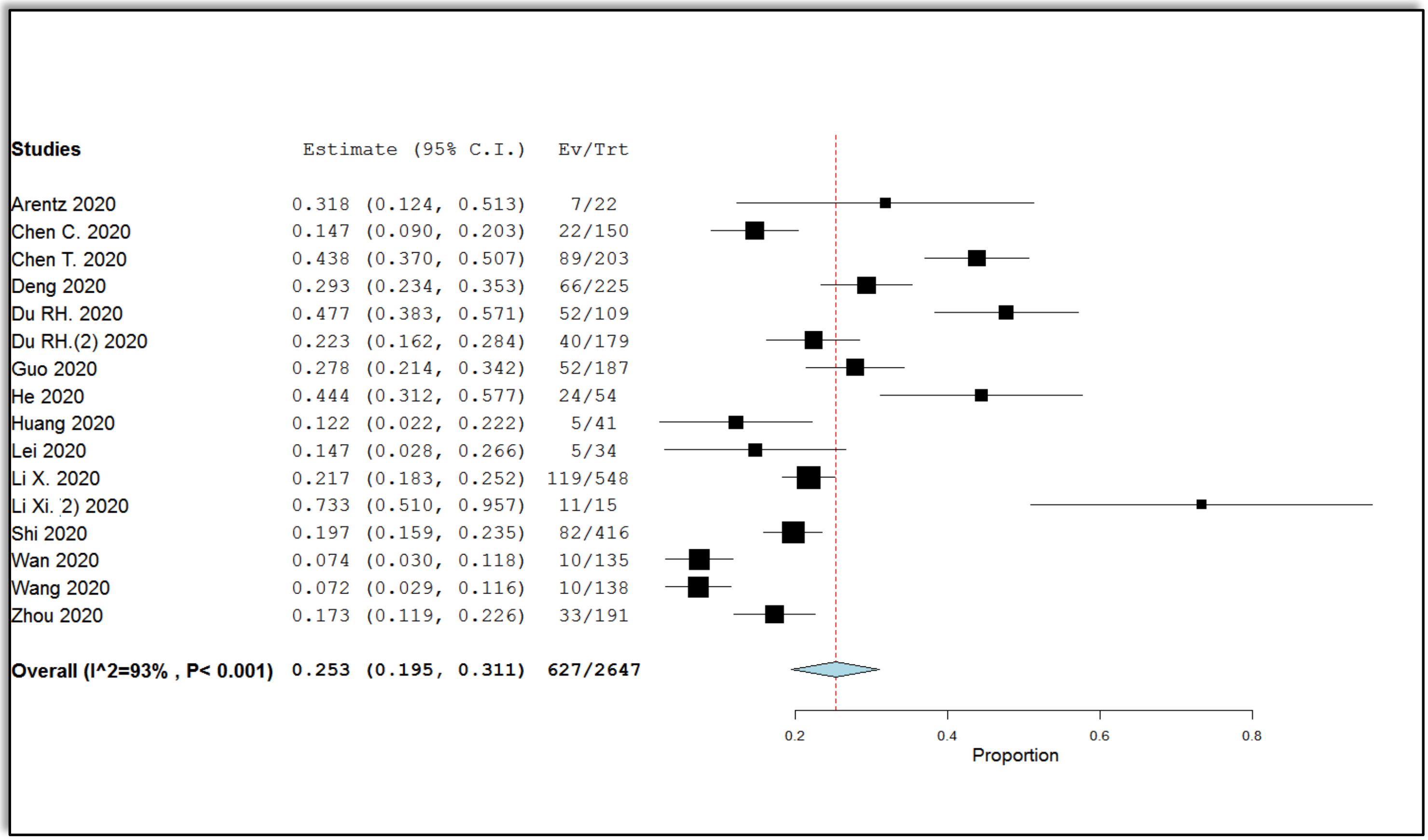

Figure 2. Forest plot of the pooled frequency analysis of newly developed arrhythmia

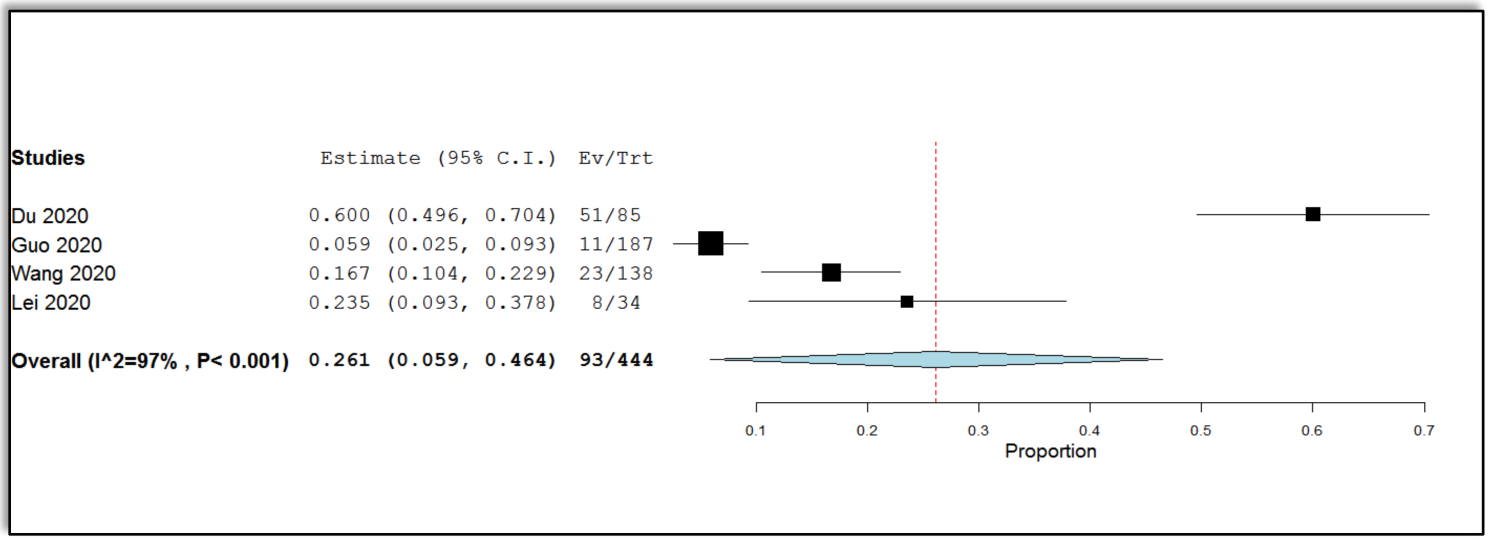

Figure 3. Forest plot of the pooled frequency analysis of newly developed heart failure

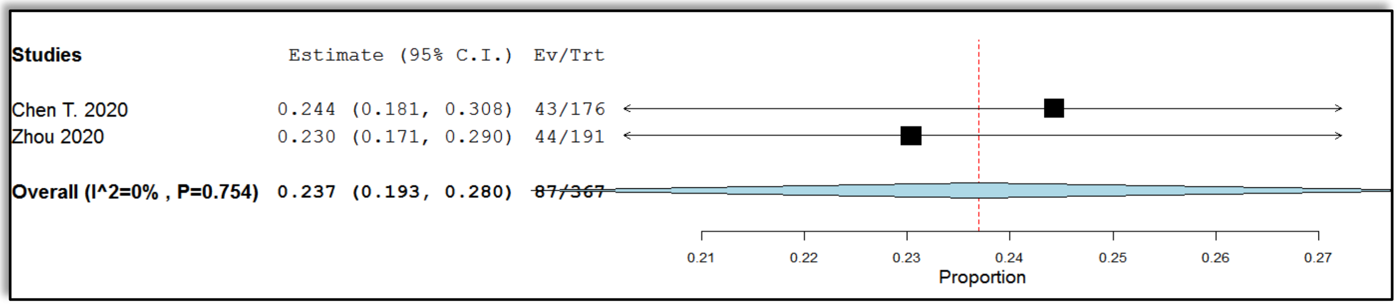

Figure 4. Forest plot of the pooled frequency analysis of chest pain or chest tightness

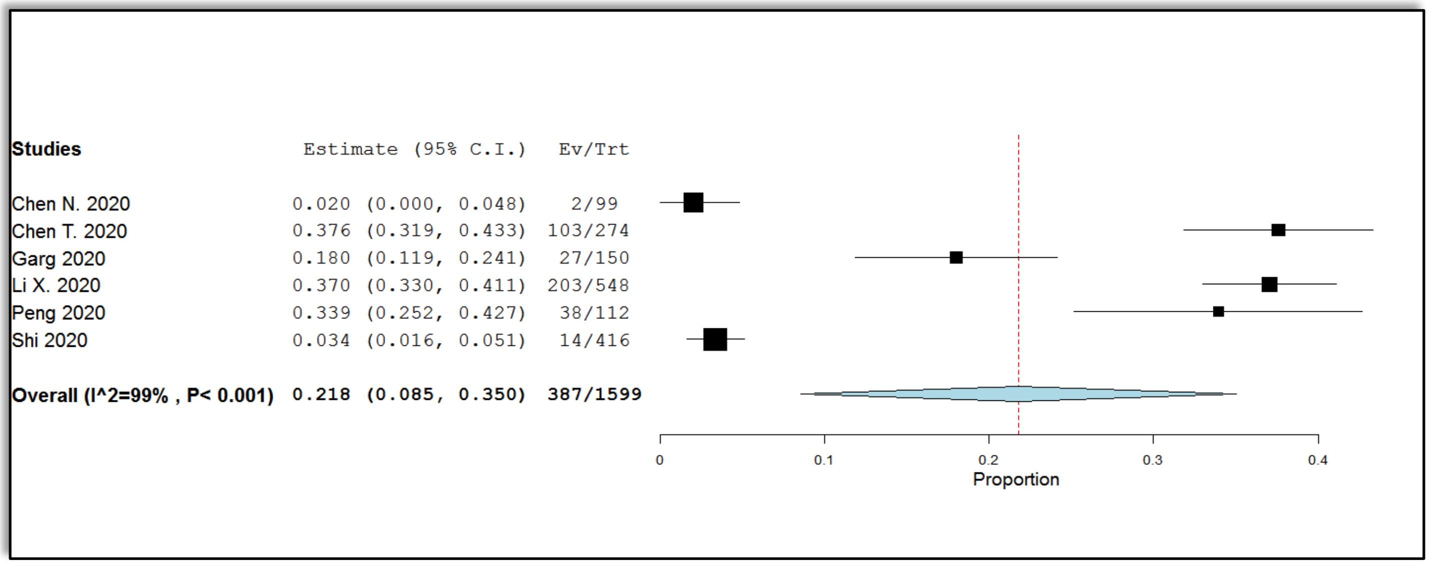

Figure 5. Forest plot of the pooled frequency analysis of palpitation

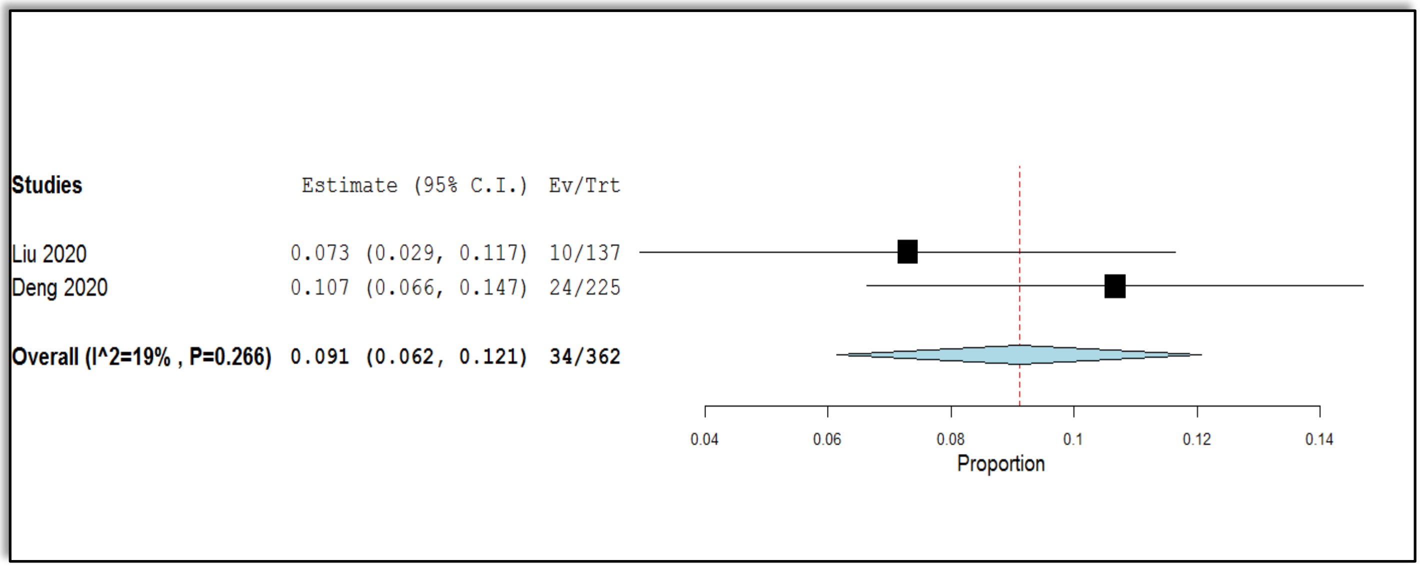

Figure 6. Forest plot of the pooled frequency analysis of hypertension

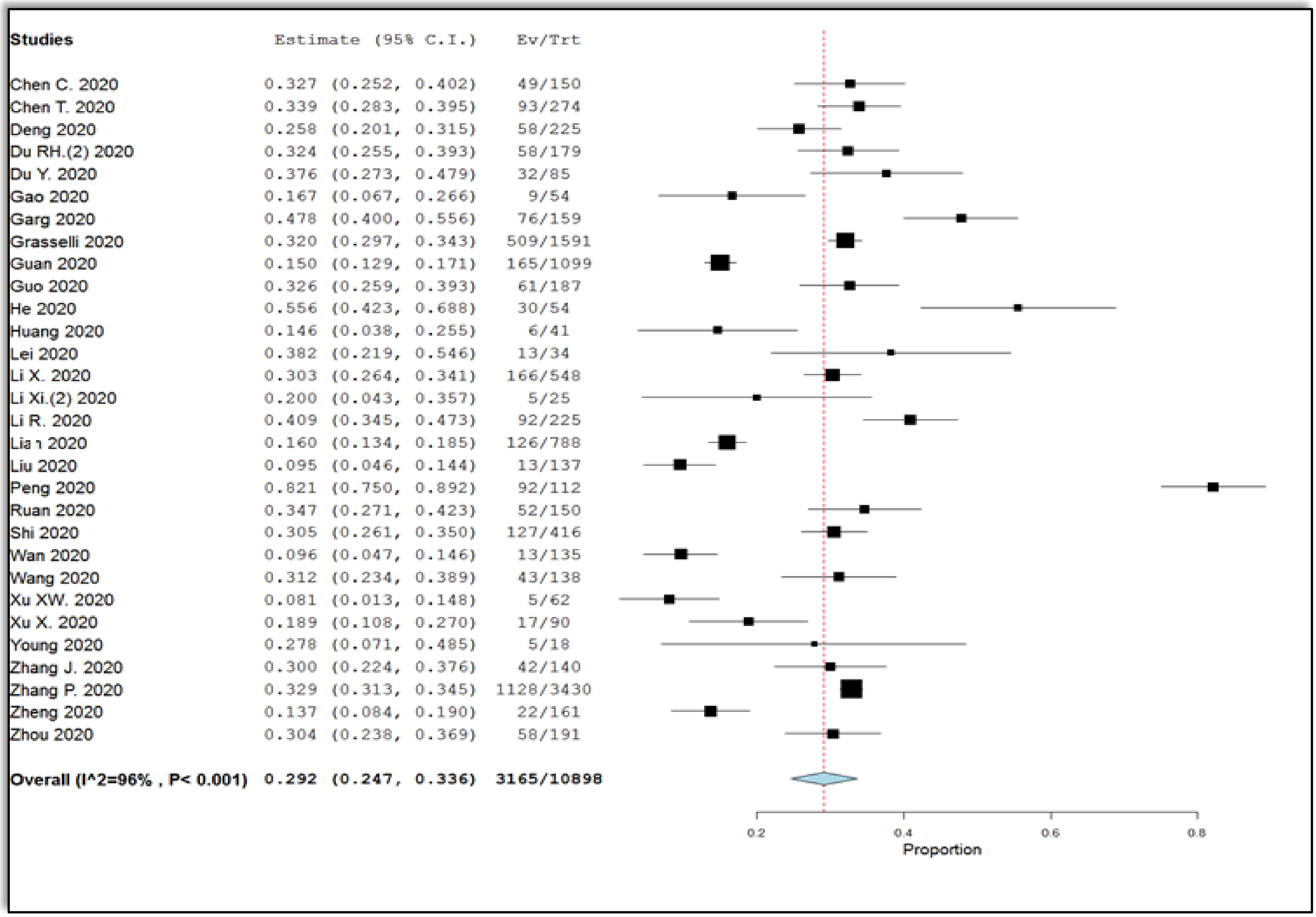

Figure 7. Forest plot of the pooled frequency analysis of pre-existing cardiovascular diseases

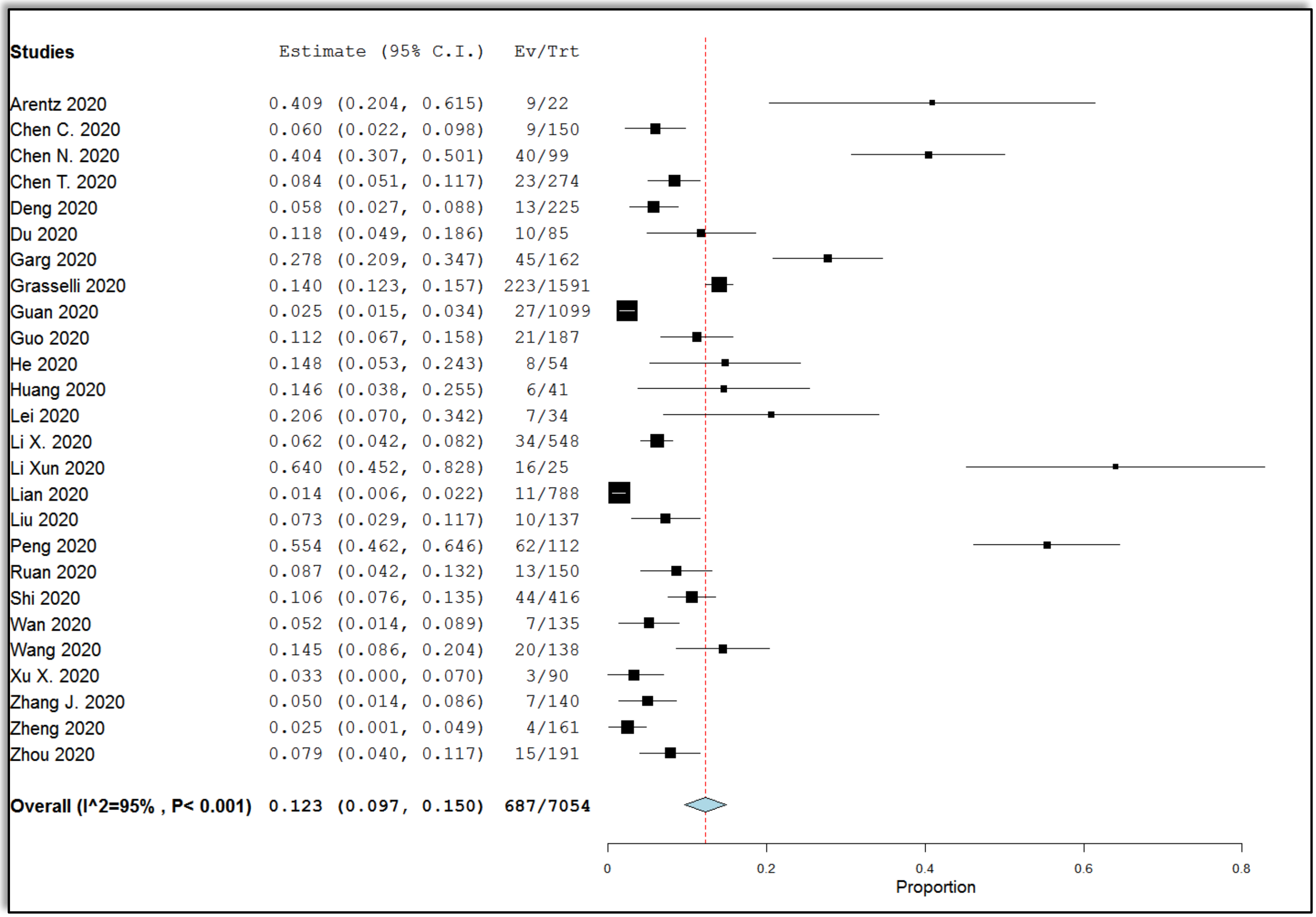

Figure 8. Forest plot of the pooled frequency analysis of pre-existing heart failure

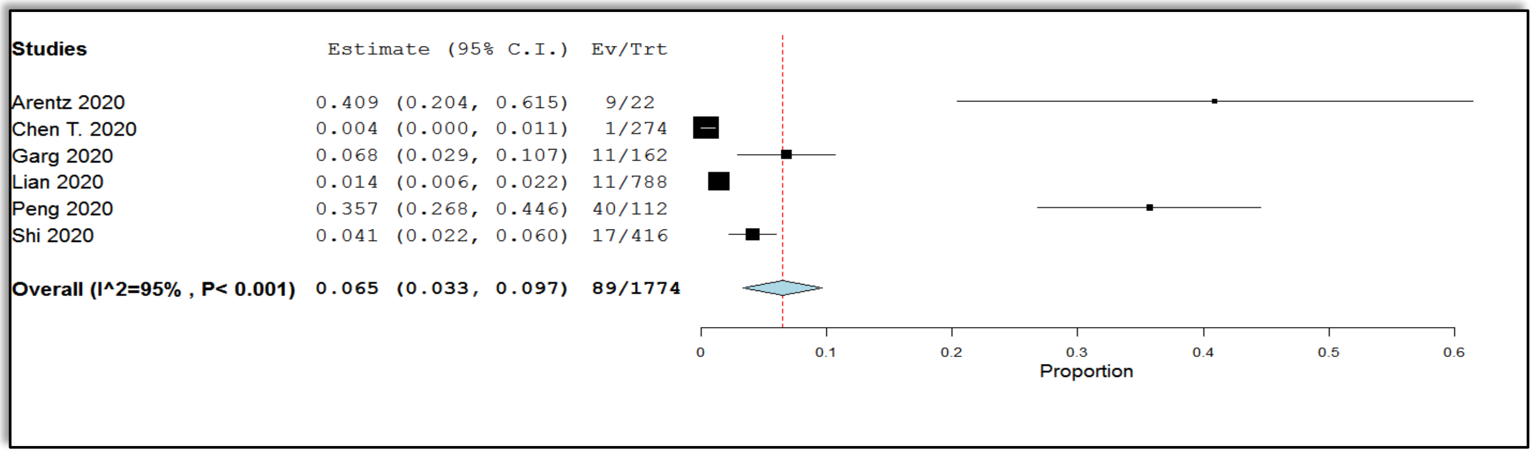

Figure 9. Forest plot of the pooled frequency analysis of pre-existing diabetes

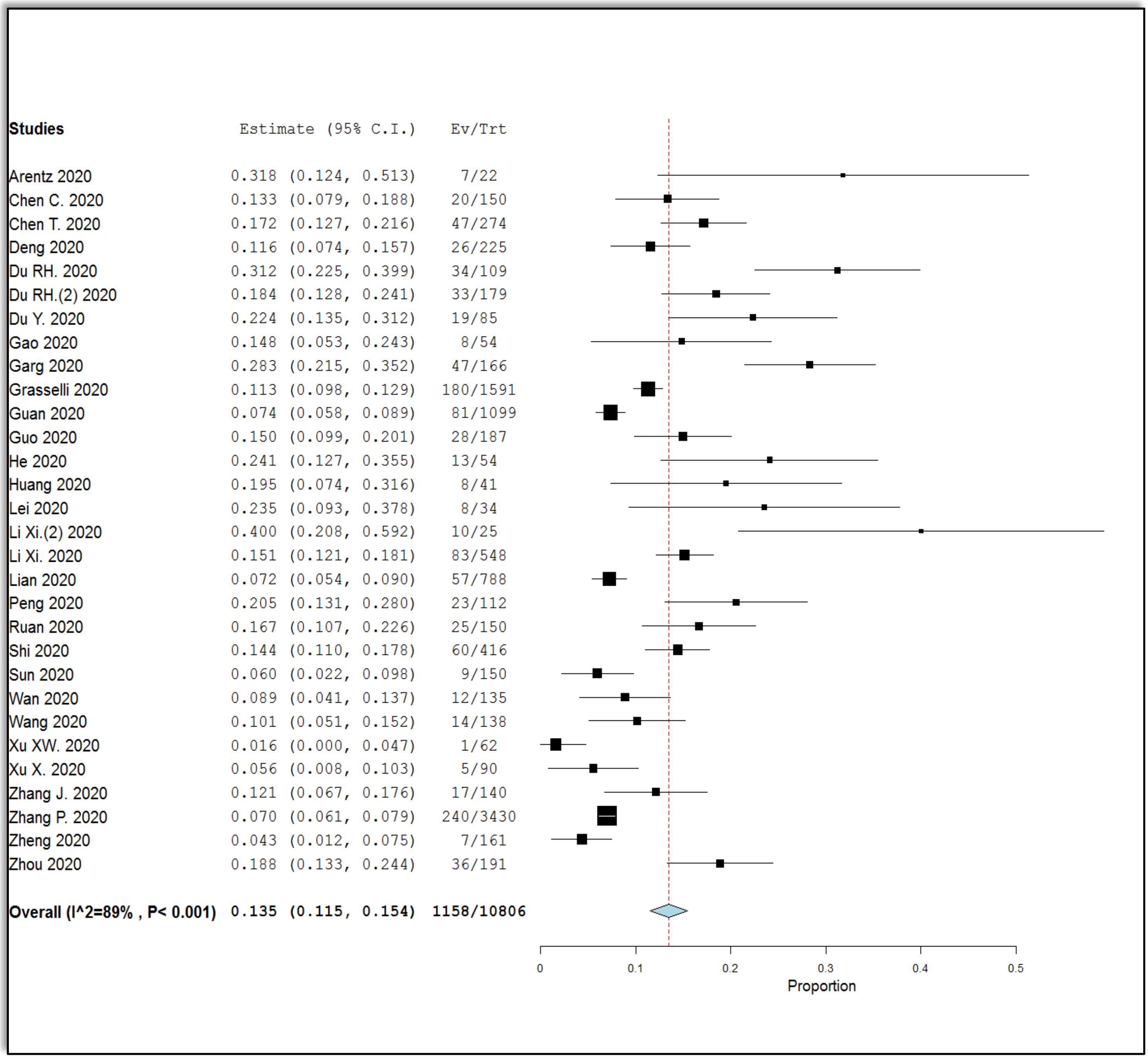

Figure 10. Forest plot of the pooled frequency analysis of patients with elevated NT-pro BNP levels

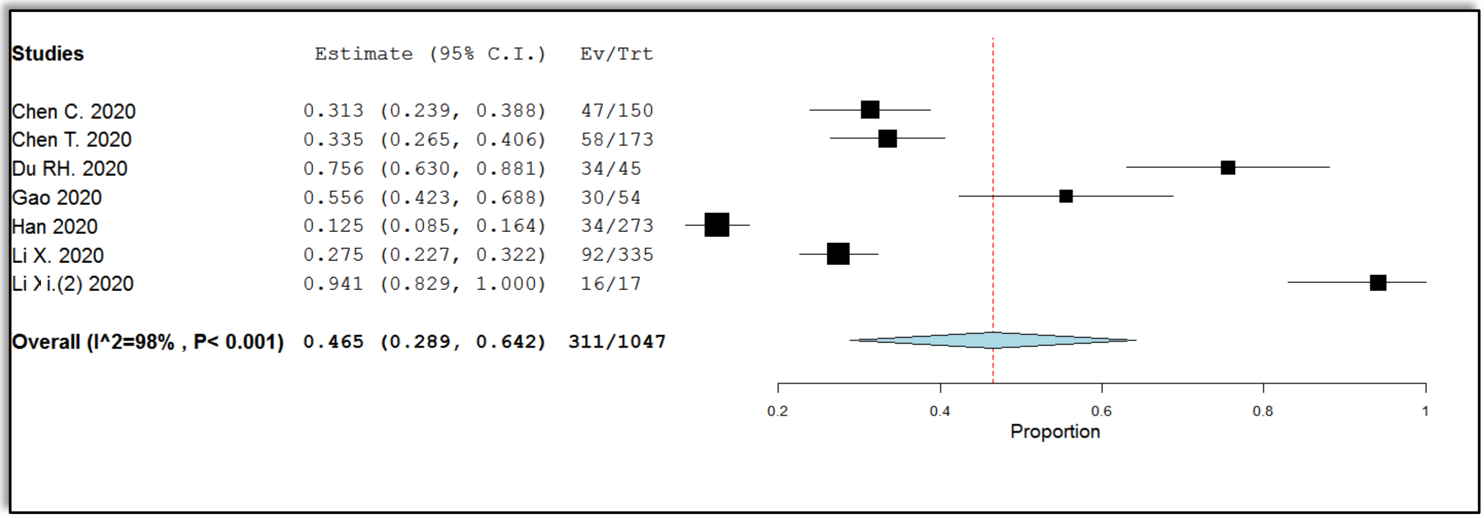

Figure 11. Forest plot of the pooled frequency analysis of patients with elevated cardiac troponins levels

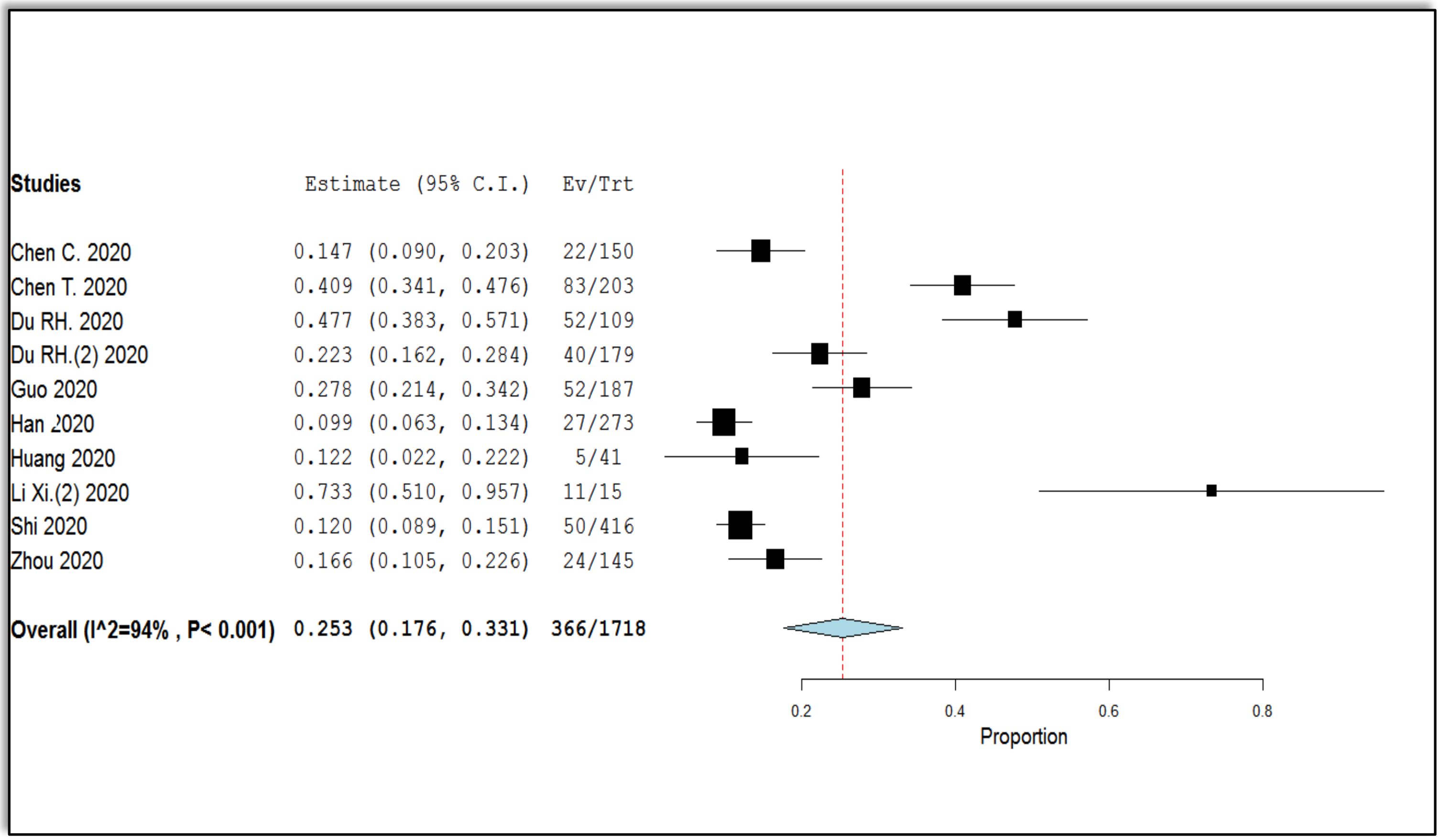

Figure 12. Forest plot of the pooled frequency analysis of patients with elevated creatine kinase-MB levels

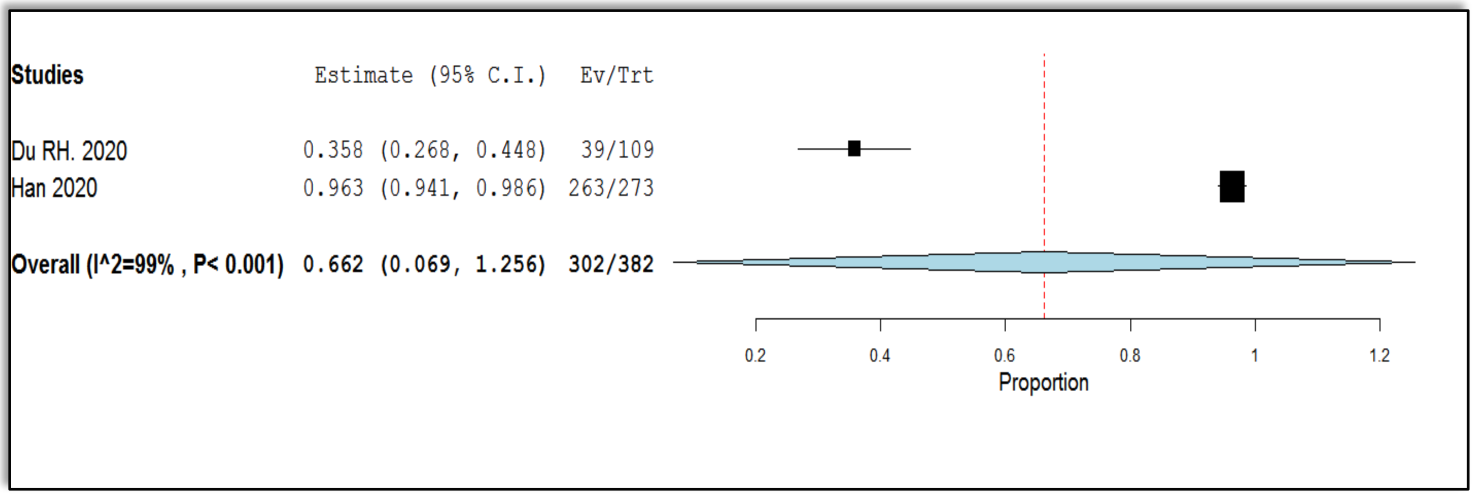

Figure 13. Forest plot of the pooled frequency analysis of patients with elevated creatine kinase levels

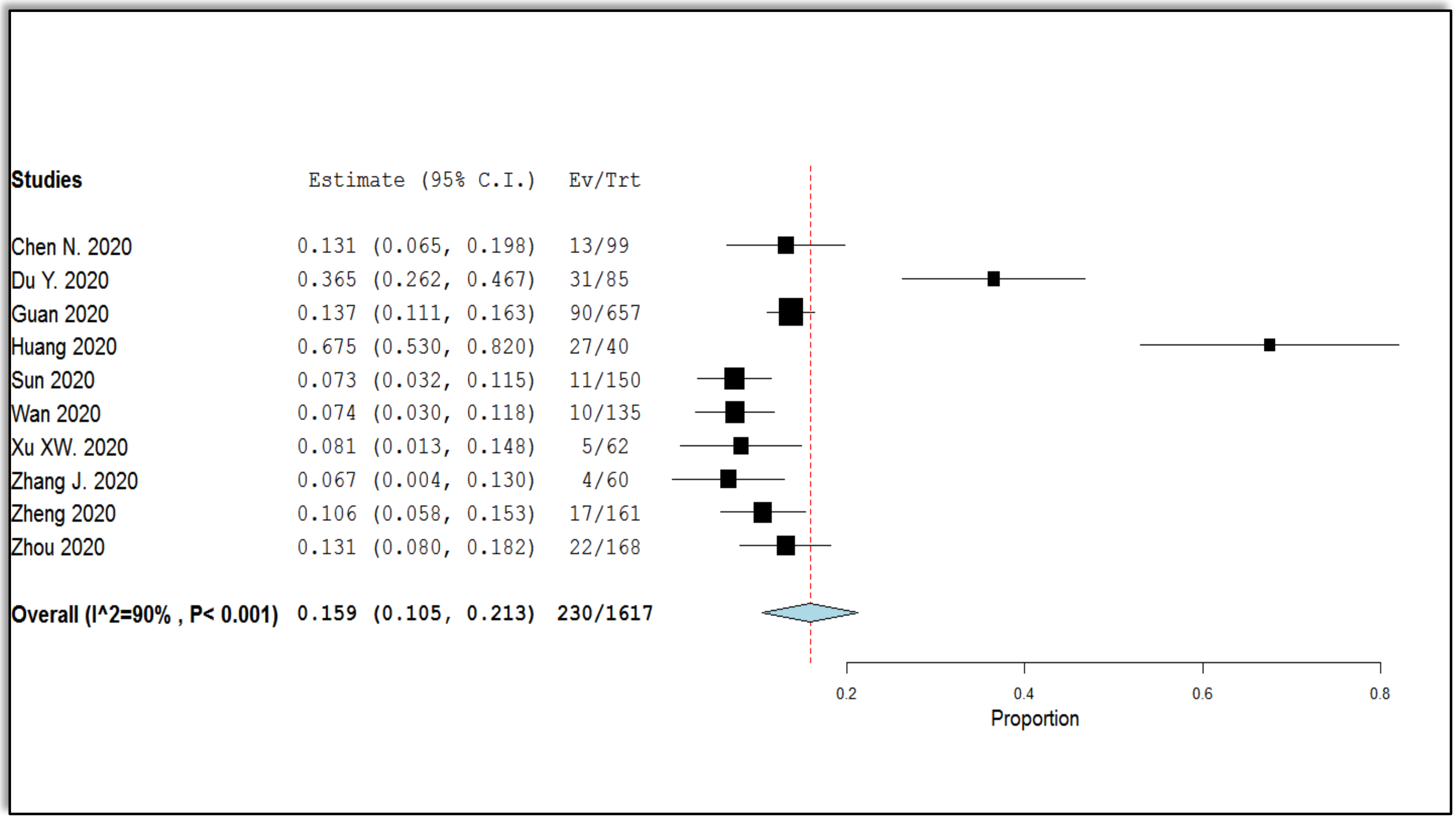

Figure 14. Forest plot of the pooled frequency analysis of patients with elevated D-dimer levels

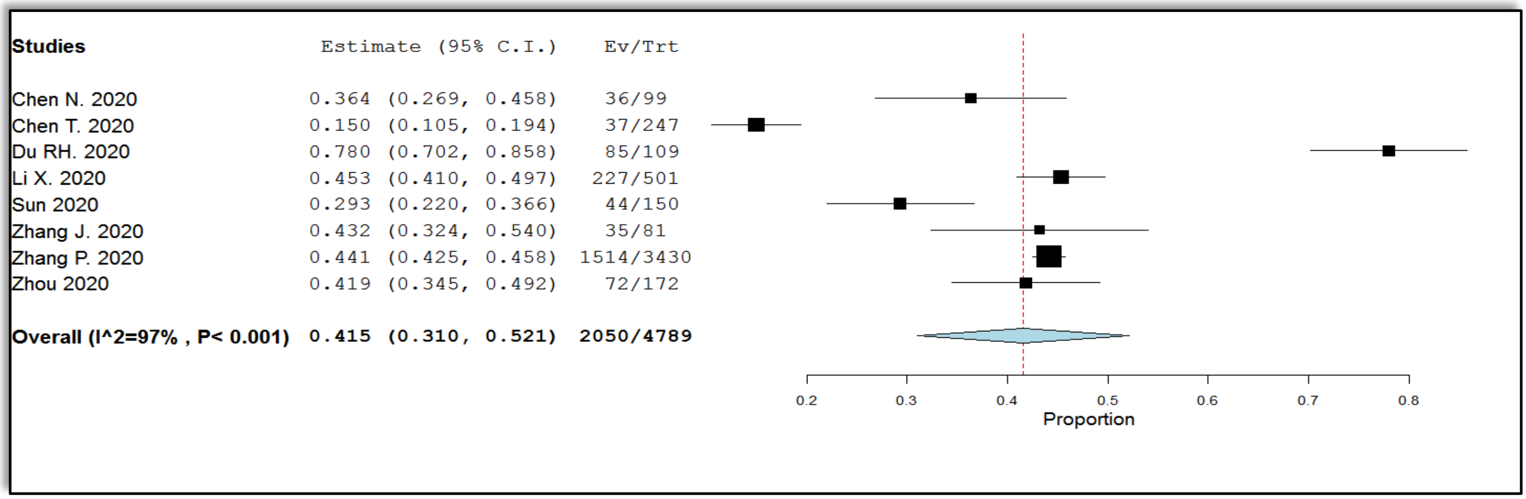

Figure 15. Forest plot of the pooled frequency analysis of patients with elevated lactate dehydrogenase levels

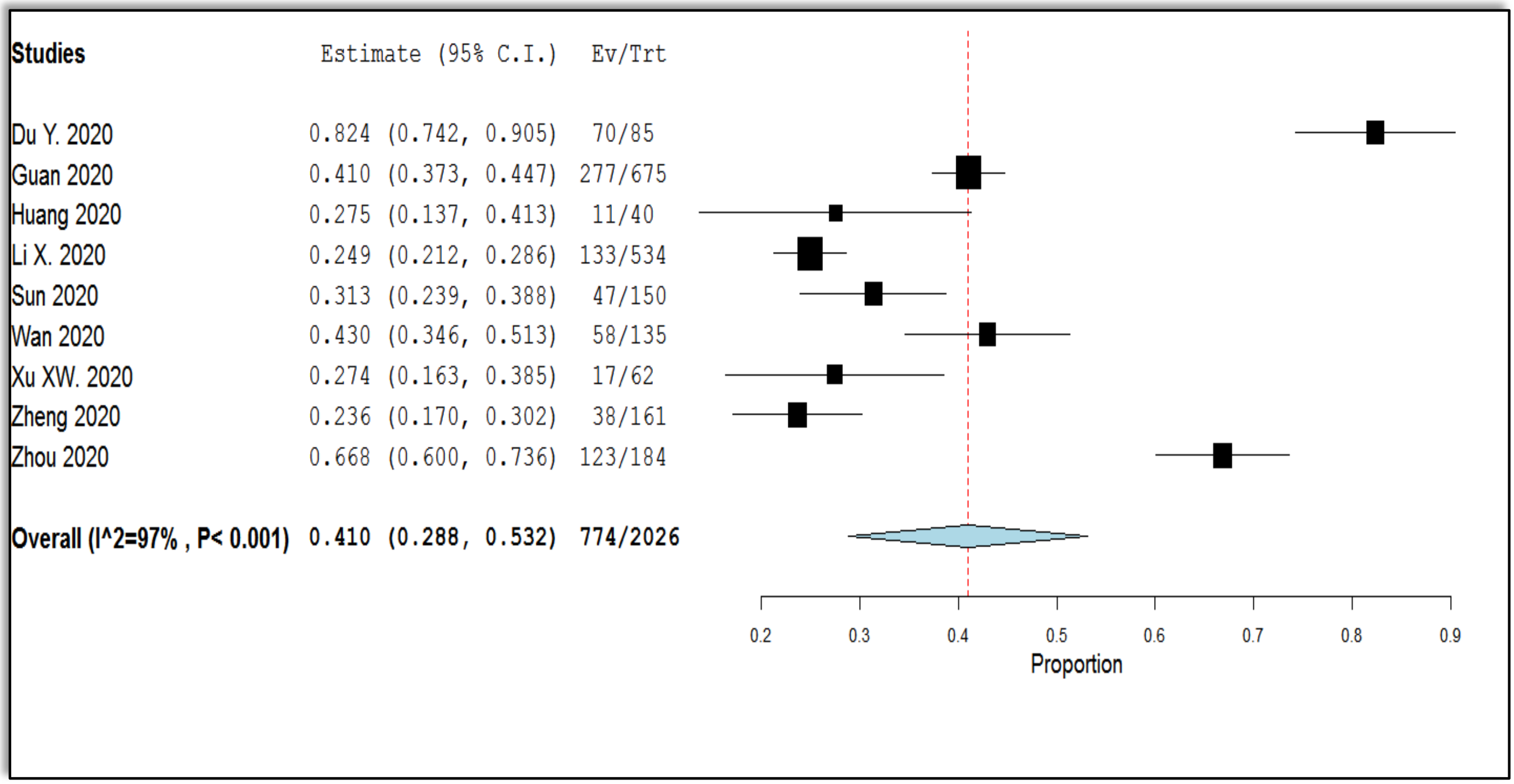

Figure 16. Forest plot of the pooled frequency analysis of patients with elevated interleukin- 6 levels

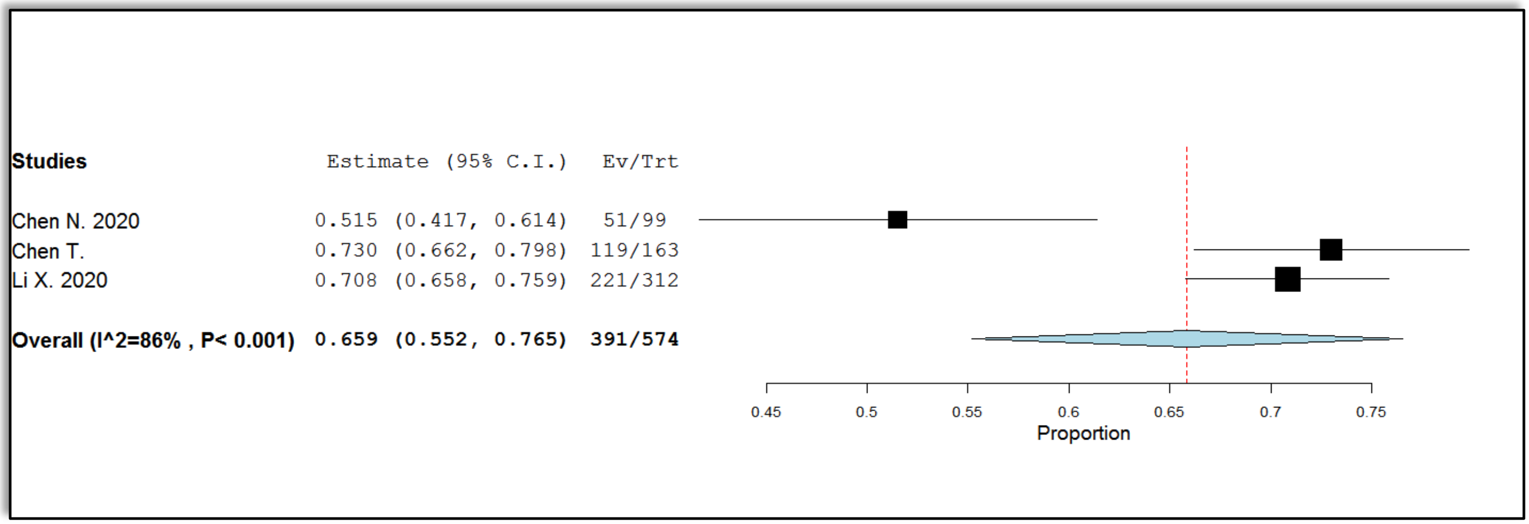

Figure 17. Forest plot of the pooled frequency analysis of patients with elevated C-Reactive Protein (CRP) levels

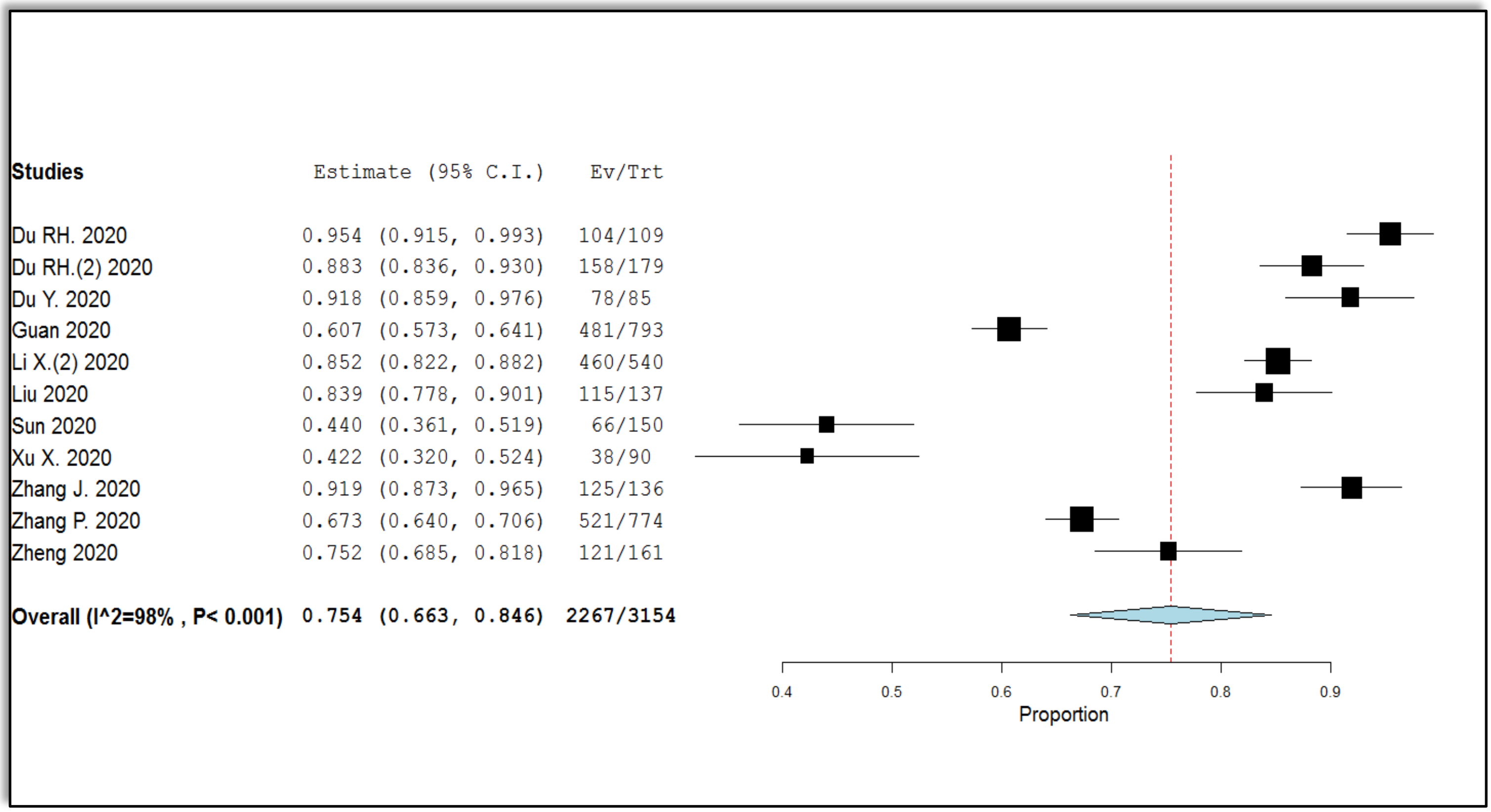

Figure 18. Forest plot of the pooled frequency analysis of patients with elevated erythrocyte sedimentation rate (ESR) levels

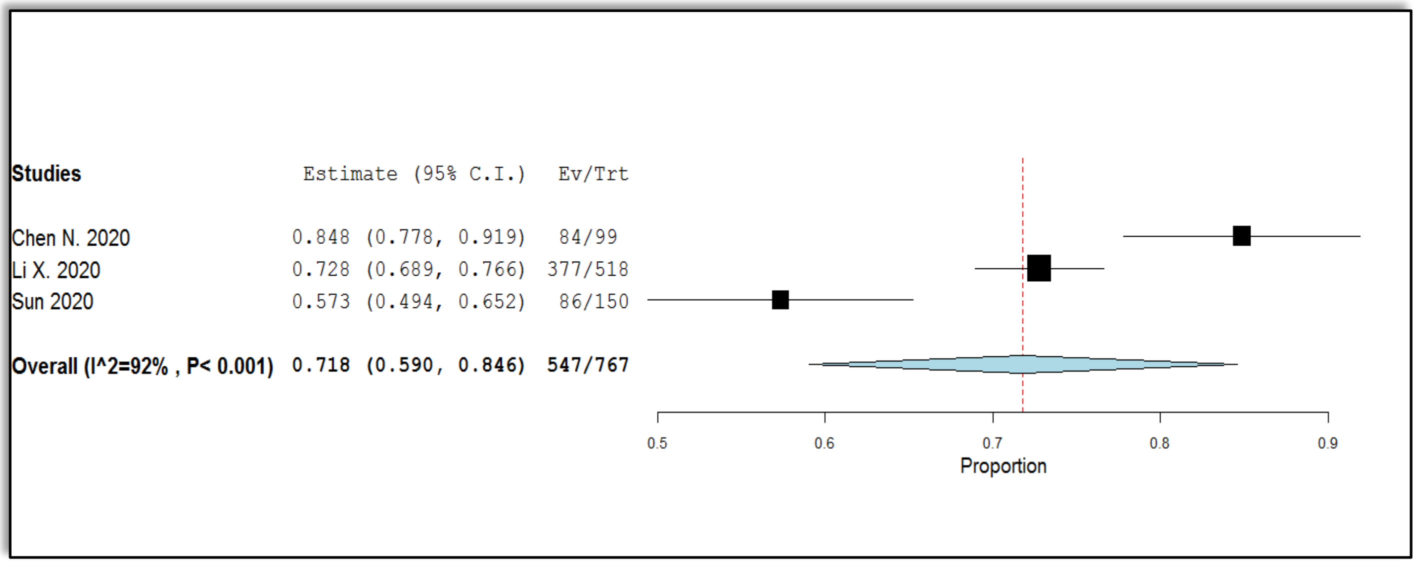

Figure 19. Forest plot of the pooled frequency analysis of patients with elevated ferritin levels

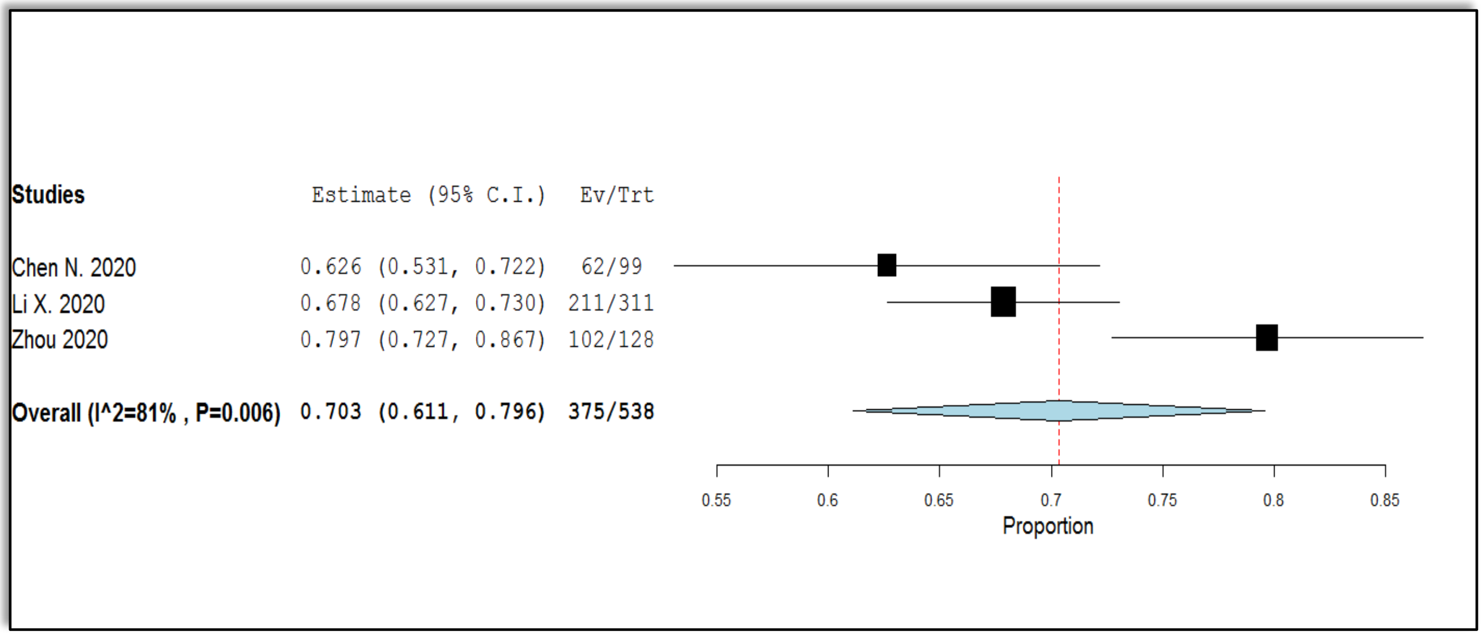

Supplement: Supplementary file 5 — Additional file 5: Supplementary Material 5 (S5) Figure 1. Forest plot of the pooled frequency analyses ( including Figure 1. Forest plot of the pooled frequency analysis of newly developed acute cardiac injury. Figure 2. Forest plot of the pooled frequency analysis of newly developed arrhythmia. Figure 3. Forest plot of the pooled frequency analysis of newly developed heart failure. Figure 4. Forest plot of the pooled frequency analysis of chest pain or chest tightness. Figure 5. Forest plot of the pooled frequency analysis of palpitation. Figure 6. Forest plot of the pooled frequency analysis of hypertension. Figure 7. Forest plot of the pooled frequency analysis of pre-existing cardiovascular diseases. Figure 8. Forest plot of the pooled frequency analysis of pre-existing heart failure. Figure 9. Forest plot of the pooled frequency analysis of pre-existing diabetes. Figure 10. Forest plot of the pooled frequency analysis of patients with elevated NT-pro BNP levels. Figure 11. Forest plot of the pooled frequency analysis of patients with elevated cardiac troponins levels. Figure 12. Forest plot of the pooled frequency analysis of patients with elevated creatine kinase-MB levels. Figure 13. Forest plot of the pooled frequency analysis of patients with elevated creatine kinase levels. Figure 14. Forest plot of the pooled frequency analysis of patients with elevated D-dimer levels. Figure 15. Forest plot of the pooled frequency analysis of patients with elevated lactate dehydrogenase levels. Figure 16. Forest plot of the pooled frequency analysis of patients with elevated interleukin- 6 levels. Figure 17. Forest plot of the pooled frequency analysis of patients with elevated C-Reactive Protein (CRP) levels. Figure 18. Forest plot of the pooled frequency analysis of patients with elevated erythrocyte sedimentation rate (ESR) levels. Figure 19. Forest plot of the pooled frequency analysis of patients with elevated ferritin levels). [file 43044_2020_75_MOESM5_ESM.pdf]
