# Supplementary material for: Cardiovascular disease in COVID-19: a systematic review and meta-analysis of 10,898 patients and proposal of a triage risk stratification tool
Source: Egypt Heart J. 2020 Jul 13;72:41. doi: 10.1186/s43044-020-00075-z (PMC7356124; doi:10.1186/s43044-020-00075-z)

**Supplementary Material 6 (S 6)**

**Figure 1. Forest plots showing odds ratio for death according to newly developed acute cardiac injury, pre-existing cardiovascular disease, hypertension and diabetes mellitus**


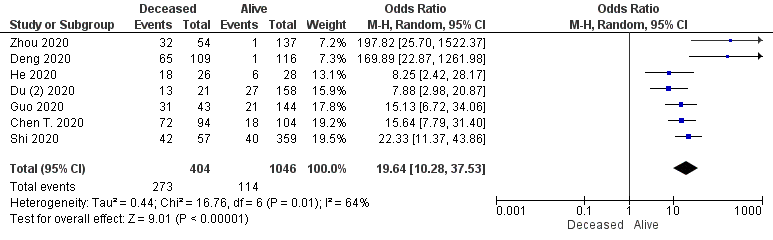


**Figure 1. Newly developed acute cardiac injury**


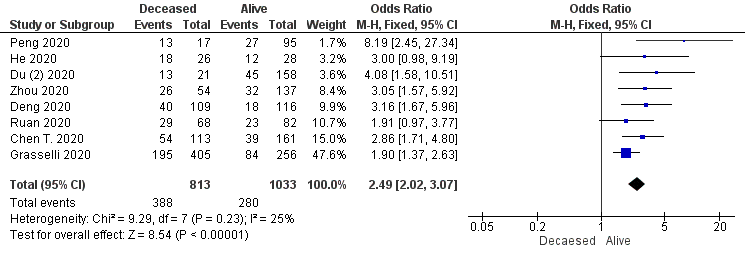


**Figure 3. Hypertension**

**Figure 2. Pre-existing cardiovascular diseases**


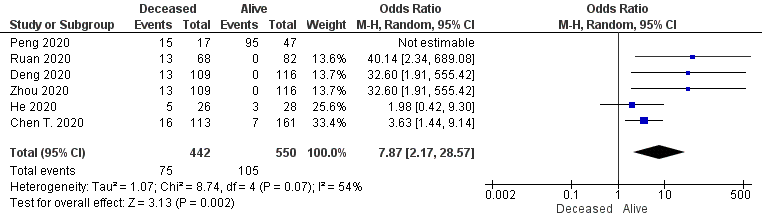


**Figure 4. Diabetes Mellitus**


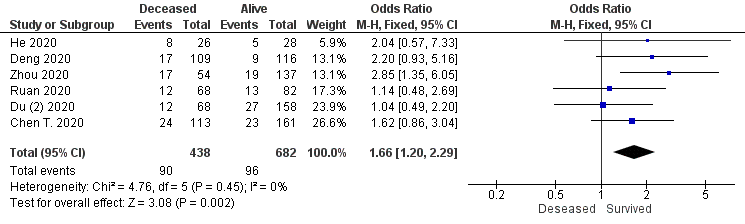

Supplement: Supplementary file 6 — Additional file 6: Forest plots showing odds ratio for death according to newly developed acute cardiac injury, pre-existing cardiovascular disease, hypertension and diabetes mellitus. [file 43044_2020_75_MOESM6_ESM.docx]
