# Supplementary material for: Cardiovascular disease in COVID-19: a systematic review and meta-analysis of 10,898 patients and proposal of a triage risk stratification tool
Source: Egypt Heart J. 2020 Jul 13;72:41. doi: 10.1186/s43044-020-00075-z (PMC7356124; doi:10.1186/s43044-020-00075-z)

Supplementary Material 7 (S7)

Figure 1. Funnel plot of studies comparing frequency of acute cardiac injury between severe or ICU group and non-severe or non-ICU group showing publication bias

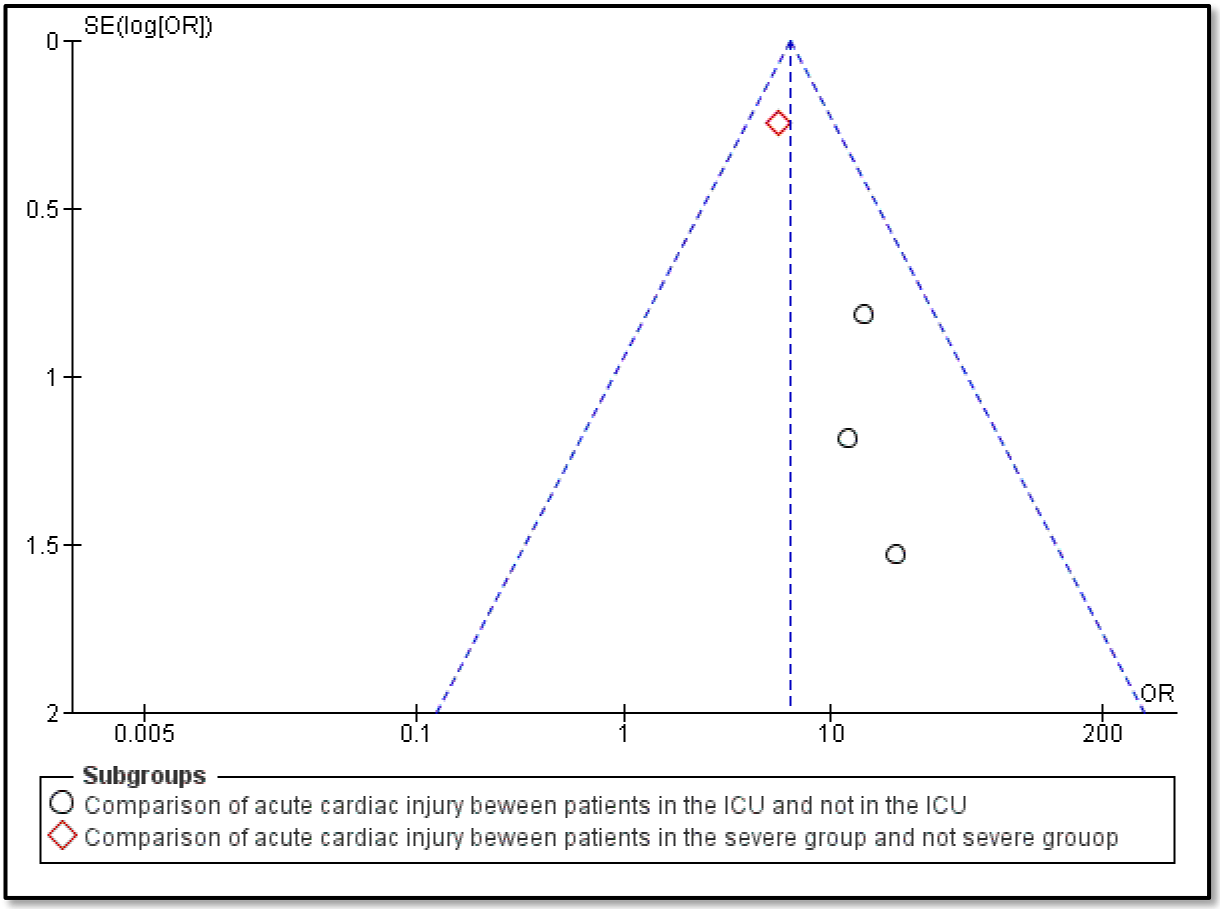

Figure 2. Funnel plot of studies comparing frequency of pre-existing cardiovascular diseases between severe or ICU group and non-sever or non-ICU group showing publication bias

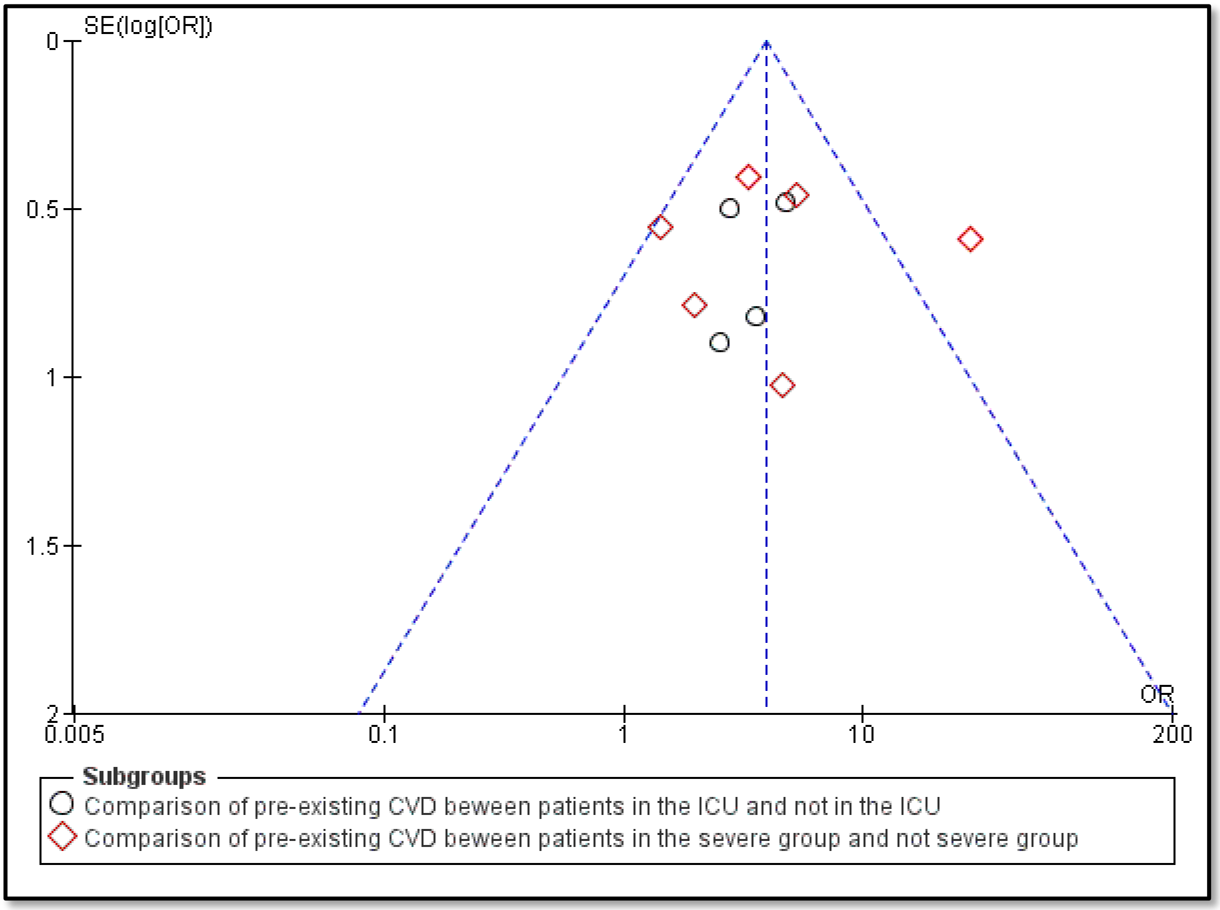

Figure 3. Funnel plot of studies comparing frequency of pre-existing cardiovascular disease between severe or ICU group and non-severe or non-ICU group showing publication bias

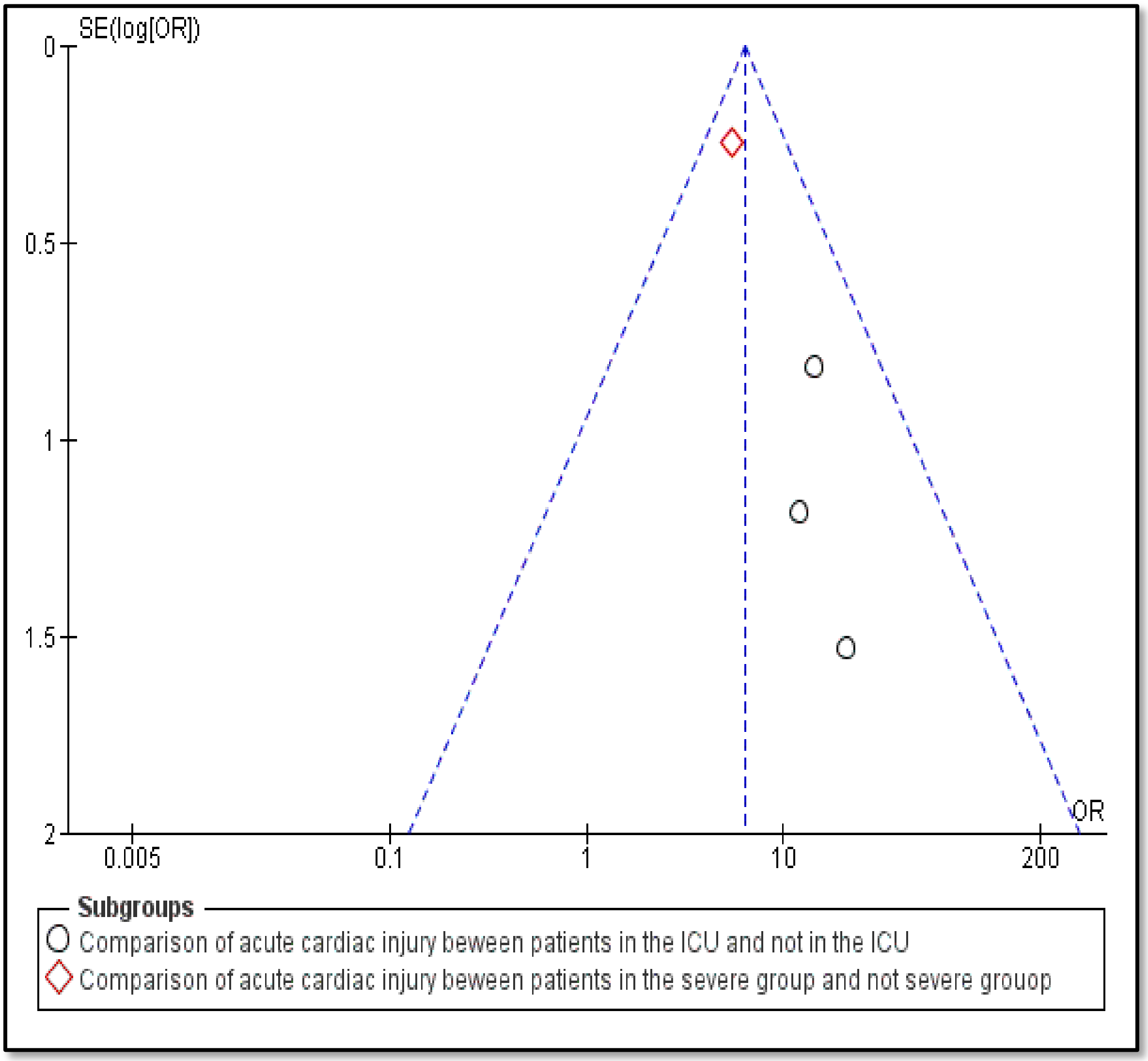

Figure 4. Funnel plot of studies comparing frequency of acute cardiac injury between deceased and survived patients showing publication bias

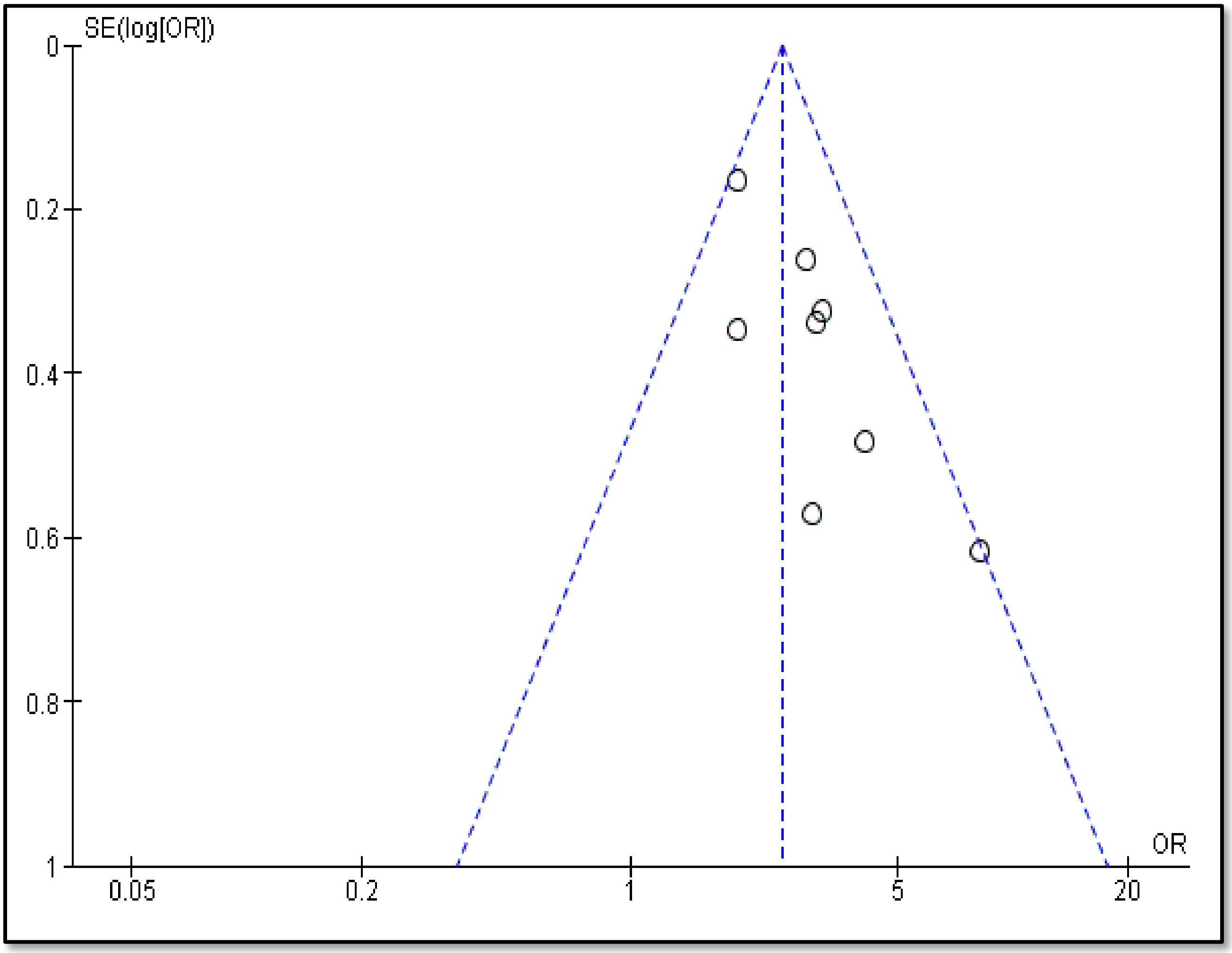

Supplement: Supplementary file 7 — Additional file 7: Supplementary Material 7 (S7) Figure 1. Funnel plot of studies comparing frequency of acute cardiac injury between severe or ICU group and non-severe or non-ICU group showing publication bias. Figure 2. Funnel plot of studies comparing frequency of pre-existing cardiovascular diseases between severe or ICU group and non-sever or non-ICU group showing publication bias. Figure 3. Funnel plot of studies comparing frequency of pre-existing cardiovascular disease between severe or ICU group and non-severe or non-ICU group showing publication bias. Figure 4. Funnel plot of studies comparing frequency of acute cardiac injury between deceased and survived patients showing publication bias [file 43044_2020_75_MOESM7_ESM.pdf]
