# Supplementary material for: Cardiovascular disease in COVID-19: a systematic review and meta-analysis of 10,898 patients and proposal of a triage risk stratification tool
Source: Egypt Heart J. 2020 Jul 13;72:41. doi: 10.1186/s43044-020-00075-z (PMC7356124; doi:10.1186/s43044-020-00075-z)
Supplement: Supplementary file 8 — Additional file 8: Supplementary Material 8 (S8) Supplementary Table 3—Cardiac pathologic findings in 13 deceased patients with COVID-19. A: absent, CAA: coronary artery atherosclerosis, CAD: coronary artery disease, ECG: electrocardiography, EM: electron microscopy, hs-cTn: high-sensitivity cardiac troponin I, LVEF: left ventricular ejection fraction, NS: not stated, P: present [file 43044_2020_75_MOESM8_ESM.docx]

**Supplementary Material 8 (S8)**

**Supplementary Table 3 – Cardiac pathologic findings in 13 deceased patients with COVID-19**

|  | Authors | country | Number of cases | Age (years) | Comorbidity | Viral cytopathic changes | Virus in the heart | Necrosis/ degeneration | Inflammatory cells | Other relevant findings | Increased  hs-cTnI | Other cardiac findings | Cause of death |
| --- | --- | --- | --- | --- | --- | --- | --- | --- | --- | --- | --- | --- | --- |
| 1 | Tavazzi et al | Italy | 1 | 69 | A | P | Present  in  interstitial cells in EM | A | Very low grade inflammatory  cells | No vasculitis or thrombosis in the Intramural vessels | NS | Fulminant myocarditis and cardiogenic shock | Recovered after VA-ECMO, but died of septic shock |
| 2 | Tian et al | China | 2 | 78  and  59 | P | A | No  EM | A | Mild focal edema | A | Present  ( 67.2 and 310.1 pg/ml; normal < 26.2) | Data regarding cardiac function is not provided. | Cardiac cause is not stated. |
| 3 | Fox et al | USA | 3 | 44 -76 | NS | A | No  EM | Scattered myocardial necrosis and atypical degeneration | Mild degree of inflammatory cells but generally not adjacent to the damaged myocytes | - Cardiomegaly - dilation of right ventricle - Normal epicardial coronary arteries | * | No clinical data regarding cardiac function is provided. | Cardiac cause is not stated. |
| 4 | Xu et al | China | 1 | 50 | A | A | No  EM | A | a few mononuclear inflammatory cells in the interstitial tissue | A | NS | No clinical data regarding cardiac function is provided. | Not intubated because of claustrophobia. He died of respiratory failure and sudden cardiac arrest |
| 5 | Barton et al | USA | 2 | 77 and 42 | P | A | No  EM | A | A | A | NS | Acute myocardial ischemia  CAD | Pre-existing CAD |
|  |  |  |  |  |  |  |  |  |  |  |  | Mild CAA | Hepatic cirrhosis |
| 6 | Sala et al | Italy | 1 | 43 | A | NS | No  EM | Limited  Focal necrosis | Diffuse infiltration of T-lymphocytes | No microvascular abnormalities | Present  Up to 135 ng/L(normal: < 14) | - Diffuse U waves on ECG - QTc: 452 msc - Decreased LVEF | Reverse Tako-Tsubo syndrome with acute lymphocytic myocarditis |
| 7 | Yao et al |  | 3 | 63 | NS |  | A | Some degeneration and necrosis of myocytes | Infiltration of few lymphocytes, monocytes and neutrophils  Edema | Infiltration of mostly Macrophage and a few CD4-positive T cells | NS | NS | Cardiac cause is not stated. |
|  |  |  |  | 69 |  |  |  |  |  |  |  |  |  |
|  |  |  |  | 79 |  |  |  |  |  |  |  |  |  |

*The authors have autopsied three of four patients. However, in the table they have defined which data belongs to autopsied patients. The troponin I level was normal in one, near normal in another, slightly increased in the third patient and more than 50 times of normal in the fourth patient.

A: absent,CAA: coronary artery atherosclerosis, CAD: coronary artery disease, ECG: electrocardiography, EM: electron microscopy, hs-cTn: high-sensitivity cardiac troponin I, LVEF: left ventricular ejection fraction, NS: not stated, P: present
